# Oral nanotherapeutic formulation of insulin with reduced episodes of hypoglycaemia

**DOI:** 10.1038/s41565-023-01565-2

**Published:** 2024-01-02

**Authors:** Nicholas J. Hunt, Glen P. Lockwood, Scott J. Heffernan, Jarryd Daymond, Meng Ngu, Ramesh K. Narayanan, Lara J. Westwood, Biswaranjan Mohanty, Lars Esser, Charlotte C. Williams, Zdenka Kuncic, Peter A. G. McCourt, David G. Le Couteur, Victoria C. Cogger

**Affiliations:** 1 Faculty of Medicine and Health, The University of Sydney, Camperdown, New South Wales Australia; 2 Sydney Nano Institute, The University of Sydney, Camperdown, New South Wales Australia; 3 Charles Perkins Centre, The University of Sydney, Camperdown, New South Wales Australia; 4 ANZAC Research Institute, Concord Repatriation General Hospital, Sydney Local Health District (SLHD), Concord, New South Wales Australia; 5 Royal Prince Alfred Hospital, SLHD, Camperdown, New South Wales Australia; 6 Sydney Business School, The University of Sydney, Camperdown, New South Wales Australia; 7 Department of Gastroenterology, Concord Repatriation General Hospital, SLHD, Concord, New South Wales Australia; 8 Sydney Analytical Core Research Facility, The University of Sydney, Camperdown, New South Wales Australia; 9 CSIRO Manufacturing, Clayton, Victoria Australia; 10 School of Physics, The University of Sydney, Camperdown, New South Wales Australia; 11 Department of Medical Biology, University of Tromsø—The Arctic University of Norway, Tromsø, Norway

**Keywords:** Nanoparticles, Drug delivery, Drug delivery

## Abstract

Injectable insulin is an extensively used medication with potential life-threatening hypoglycaemic events. Here we report on insulin-conjugated silver sulfide quantum dots coated with a chitosan/glucose polymer to produce a responsive oral insulin nanoformulation. This formulation is pH responsive, is insoluble in acidic environments and shows increased absorption in human duodenum explants and *Caenorhabditis elegans* at neutral pH. The formulation is sensitive to glucosidase enzymes to trigger insulin release. It is found that the formulation distributes to the liver in mice and rats after oral administration and promotes a dose-dependent reduction in blood glucose without promoting hypoglycaemia or weight gain in diabetic rodents. Non-diabetic baboons also show a dose-dependent reduction in blood glucose. No biochemical or haematological toxicity or adverse events were observed in mice, rats and non-human primates. The formulation demonstrates the potential to orally control blood glucose without hypoglycaemic episodes.

## Abstract (web summary)

Insulin injections are not ideal and have an increased risk of hypoglycaemia. A preferable oral formulation based on silver sulfide quantum dots coated with a chitosan/glucose polymer is discussed, which has controlled insulin release and reduced risk of hypoglycaemia, and demonstrates applications in rodent and non-human primate models.

## Main

The escalating disease burden of diabetes mellitus, with a global prevalence of 425 million people, is a critical health priority with multifaceted demands for patients, carers, health systems and the economy^1^. Of this population, 75 million are insulin dependent^1,2^. These severe insulin-deficient and insulin-resistant subtypes require strict glycaemic control via a combination of injectable or infusions of insulin, lifestyle interventions and continuous glucose monitoring to reduce the incidence of acute adverse events (hyperglycaemia and hypoglycaemia) and the development of long-term complications such as cardiovascular disease^3^ or nephro-, neuro- and retinopathies^4–7^.

Of these complications, hypoglycaemia is a dangerous side effect of insulin treatment^8^, correlating with reduced health-related quality of life and productivity and increased health-care resource utilization^9–11^. In 2020, the economic cost of type 1 diabetes (T1D) alone was 2.9 billion USD, with 60% due to indirect costs (patient well-being and productivity)^12,13^. Reduction in hypoglycaemic events without the loss of glycaemic control is critical to improve patient well-being^14^ and reduce the risk of cardiovascular disease and mortality^8^. In light of these costs and existing injectable insulin complications, there is a need to examine alternative methods of insulin delivery to redesign insulin to end hypoglycaemic risk^15^. These technologies must also be cost-effective given the recent evidence that insulin pump management is not a health economic cost-saving measure compared with user education^16^.

Oral insulin provides a solution to these challenges, as it is considered to mimic the endogenous release of insulin^17^. Most insulin secreted by the pancreas acts on the liver, and only a small fraction avoids hepatic clearance and acts systemically^17^. Likewise, after oral ingestion, oral insulin is first delivered to the liver via portal circulation^17^. However, previous clinical trials of oral insulin formulations have highlighted a minimal effect on reducing the incidence of hypoglycaemia^18^. Oral insulin also presents several advantages to long-term adherence and acceptability of medications as insulin injections can be especially challenging for children or older people and newly diagnosed insulin-dependent type 2 diabetes, resulting in avoidance, hesitancy or delay of insulin therapy^19,20^. Additionally, injectable insulin is not considered a viable treatment in new therapeutic areas (such as cognitive impairment and Alzheimer’s disease)^21^, where it may have clinical efficacy. No oral insulin is available in the market, but proof of translation is provided with oral semaglutide^22^.

Previously, we have shown that silver sulfide (Ag_2_S) quantum dots (QDs) are orally bioavailable and increase the rate of absorption, oral bioavailability and hepatocyte targeting of metformin and nicotinamide mononucleotide^23,24^. These drugs are already orally bioavailable; however, conjugation with QDs increased the potency 100–10,000-fold^23,24^ and overcame age-associated impaired drug efficacy^25–27^. Here we applied this nanotechnology platform to insulin in combination with a pH- and enzyme-sensitive encapsulating polymer^28^.

## Nanocarrier is pH sensitive

**Fig. 1:**
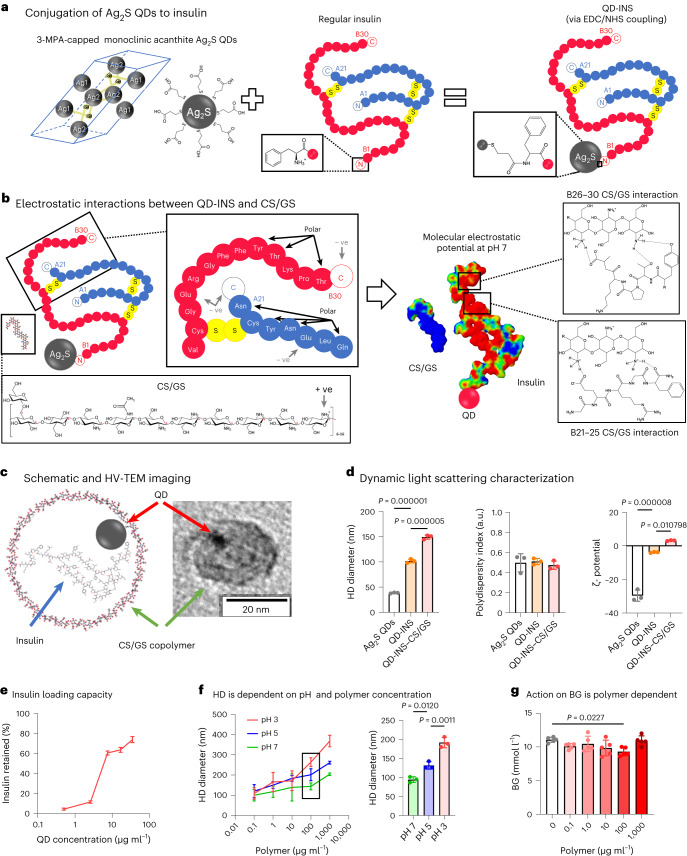
Formulation and characterization of oral insulin (QD-INS–CS/GS). **a**, Schematic of the formulation illustrating the attachment of insulin via the N terminus to 3-MPA-capped Ag_2_S QDs by EDC/NHS chemical coupling. **b**, Schematic of the formation for the attachment of CS/GS via electrostatic interactions with QD-INS. At pH 7, QD-INS has a negative *ζ*-potential (Extended Data Fig. 1) concentrated at the C terminus of the α and β chains. CS/GS has a positive *ζ*-potential (Extended Data Fig. 1) and interacts via hydrogen bonding between the primary amines of CS/GS and B21, 25–27 and 30 amino acids. Conjugation of insulin with QD demonstrates similar FTIR spectra to insulin alone; coating with CS/GS modified the FTIR spectra to appear as a CS/GS alone (Extended Data Fig. 2a). **c**, HV-TEM imaging shows the spherical 20 nm nanoparticles containing Ag_2_S QDs. **d**, Dynamic light scattering measurements (HD diameter, polydispersity index and *ζ*-potential) showing Ag_2_S QDs alone, QD-INS and QD-INS–CS/GS. The HD diameter increased with insulin and CS/GS attachment and the *ζ*-potential approaches 0 with insulin and CS/GS attachment. **e**, Percentage of insulin retained following attachment was dependent on the QD concentration; increasing the QDs (more binding sites) promoted greater insulin retainment. **f**,**g**, HD diameter was observed to increase two and three times with lower pH of 5 and 3, respectively, for QD-INS coated with 100 µg ml^–1^ CS/GS. C57BL/6 mice pre-treated with QD-INS (20 IU kg^–1^) coated with increasing polymer concentrations demonstrated reduced fasting BG only at 100 µg ml^–1^ CS/GS concentration (**f**). Data points, mean ± s.d. shown (*n* = 3 independent batches (**d**–**f**); *n* = 5 biologically independent animals (**g**) and one-way ANOVA with Tukey’s correction; *α* = 0.05).

**Extended Data Fig. 1:**
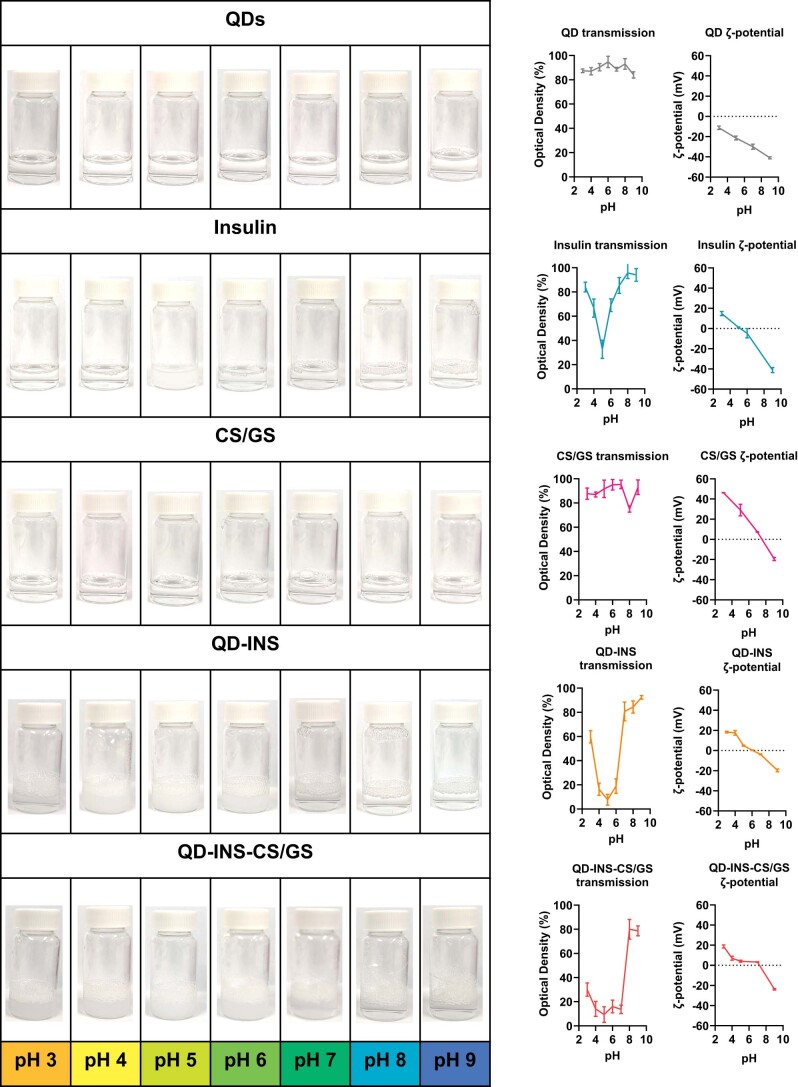
Appearance, solubility and zeta (ζ)-potential of QDs, insulin, chitosan/glucose (CS/GS), QD-INS and QD-INS/CS/GS at pH 3 to 9. 1 mg/ml solutions (5 mL total volume) were prepared for each solution. pH was adjusted to pH 3 with 1 M HCl using a pH meter. Images were collected as the pH was raised by the addition of 1 M NaOH. Images show the product solution at each pH. QDs and CS/GS demonstrated no change in solution at any pH (CS/GS at 10 mg/ml demonstrated precipitate formation at pH 8–9). Insulin showed insolubility at pH 5 only (insulin also showed insolubility with the addition of pH 7 MQ water to insulin powder, 0.01 M HCl is required for insulin solubility). QD-INS and QD-INS–CS/GS were highly insoluble at pH 4, 5 and 6. QD-INS–CS/GS was insoluble at pH 3–7. Solubility was determined by optical density of 1 mg/ml samples as pH was increased from 3 to 9. Data expressed as the mean ± s.d. (n = 3 independent batches). ζ-potential of each sample was measured using DLS. Data expressed as the mean ± s.d. (n = 3 independent batches). A ζ-potential of 0 is a neutral charged material and the point of 0 intersection is the isoelectric point. It was observed that the isoelectric point correlated with reduced solubility and optical transmission. It was observed that if ζ-potential had a broad range of pH values that were close to 0, then the solution was insoluble for these pH values.

**Extended Data Fig. 2:**
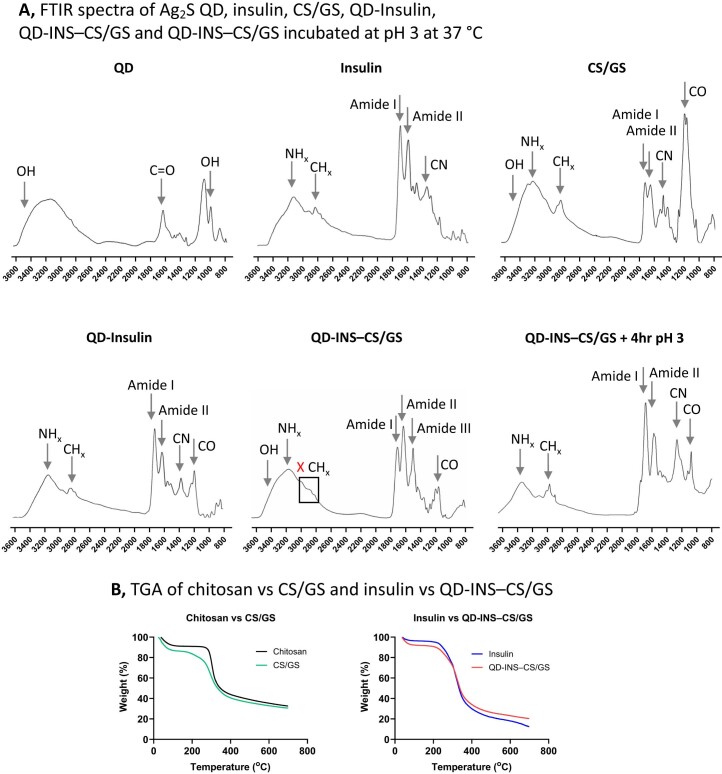
Fourier transformed infrared (FTIR) microscopy and thermogravimetric analysis (TGA) of QD-INS/CS/GS. (**a**) FTIR spectra were collected from 10 mg of dried insulin, CS/GS, QD-INS and QD-INS–CS/GS. FTIR spectra demonstrated similar characteristics between QD-INS with insulin alone and QD-INS–CS/GS with CS/GS alone. This illustrates that insulin bound to the surface of QD-INS with the addition of CS/GS producing a product with a greater similarity to CS/GS alone than insulin. Incubation QD-INS–CS/GS at pH 3 for 4 h removed CS/GS and produced spectra similar to QD-INS. (**b**) TGA data were collected from 2 to 3 mg of dried chitosan, CS/GS, insulin and QD-INS–CS/GS. TGA data demonstrate CS/GS differs from chitosan alone with greater mass loss (4.7%) at 50 °C, a new mass loss of 7.6% at 215 °C and similar mass loss with chitosan at 310 °C (40% vs 49%, CS/GS vs chitosan, respectively). QD-INS–CS/GS was observed to differ from insulin alone with 6.4% greater mass loss before 306 °C, likely due to CS/GS and an 8% reduction in mass under 700 °C likely the unreacted Ag_2_S content with residual CS/GS.

The initial challenge for oral delivery of insulin is its high solubility and degradation at low pH (Extended Data Fig. 1)^29,30^. Here we found that the attachment of insulin to Ag_2_S QDs reduced the solubility of QD insulin (QD-INS) in low-pH environments compared with insulin or QDs alone. Soluble insulin (pH 3) was bound to the surface of Ag_2_S QDs [diameter, 6.5 ± 1.2 nm (measured by transmission electron microscopy (TEM))] capped with 3-mercaptopropionic acid (3-MPA) using *N*-(3-dimethylaminopropyl)-*N*′-ethylcarbodiimide hydrochloride (EDC) and sulfo-*N*-hydroxysuccinimide (NHS) chemical crosslinking (Fig. 1a). The attachment of insulin was performed at pH 9, producing a soluble QD-INS solution. Following dialysis, a soluble QD-INS solution was present at pH 7 (Extended Data Fig. 1). Fourier transform infrared (FTIR) spectroscopy showed that QD-INS had similar peaks to insulin alone, indicative of attachment, following the removal of free insulin during dialysis (Extended Data Fig. 2a).

**Extended Data Fig. 3:**
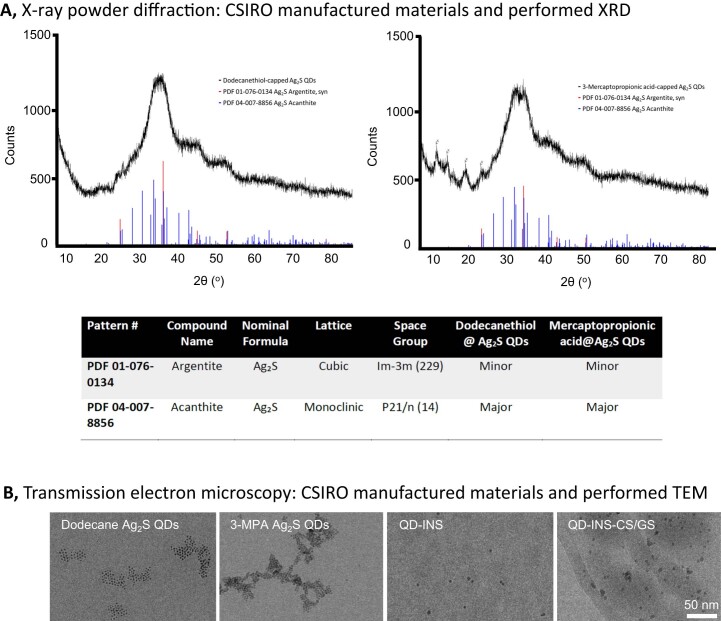
Characterization of CSIRO manufactured QD-INS–CS/GS. (**a**) XRD was performed to determine the crystallographic structure and the chemical composition of the QDs with sampling from 2 to 3 independent batches. The best match phases from the ICDD-JCPDS powder diffraction database are listed in the table and showed QDs were acanthite with a monoclinic lattice. (**b**) Representative TEM images of QDs, QD-INS, and QD-INS–CS/GS captured from a pH 4 solution are shown with scale bars showing 50 nm. Experiment was reproduced from 2 to 3 independent batches.

Validation of our QD formulation was performed by an accredited manufacturing organization, namely, the Commonwealth Scientific and Industrial Research Organisation (CSIRO) R&D Laboratory. CSIRO produced nanocrystalline monoclinic 5.5 ± 0.9 nm Ag_2_S QDs composed of acanthite (85%) with minor argentite (15%) composition. No silver metal was observed as measured by X-ray powder diffraction (Extended Data Fig. 3a,b).

QD-INS was insoluble with reduced optical density and transmission between pH 3.5 and pH 6.5 (Extended Data Fig. 1). Given that the isoelectric point of Ag_2_S QDs and insulin were 2.8 and 5.3, respectively, we investigated the changes in zeta (*ζ*)-potential of QD-INS. Between pH 3 and pH 6, the *ζ*-potential demonstrated a near-zero value, suggesting a broad range over which QD-INS maintained a net neutral charge leading to poor colloidal stability and precipitate formation (Extended Data Fig. 1).

**Extended Data Fig. 4:**
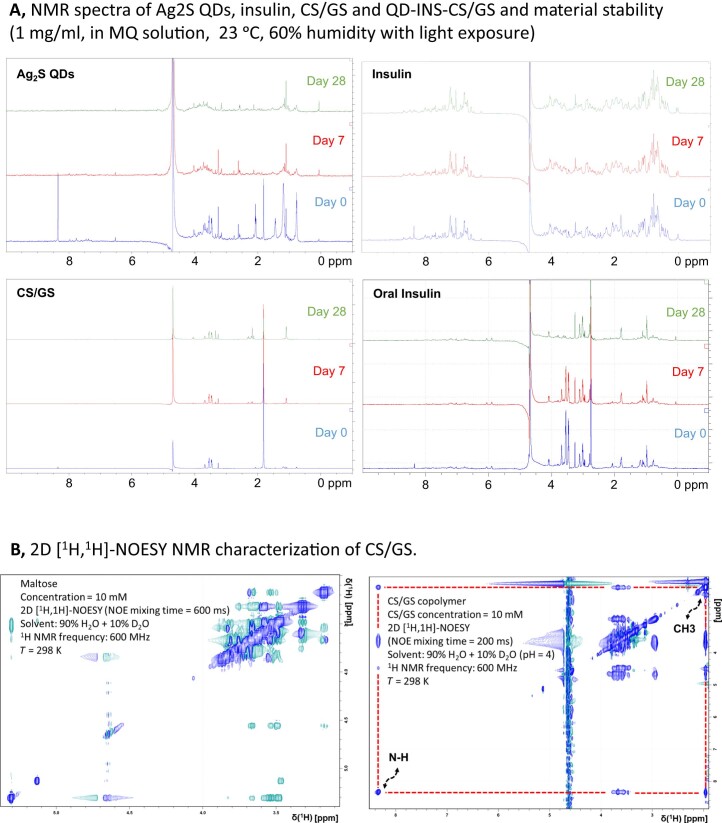
NMR characterization and stability of QD-INS–CS/GS and 2D [^1^H,^1^H]-NOESY NMR characterization of CS/GS. (**a**) NMR spectra demonstrated a similar effect to FTIR. Oral insulin (QD-INS–CS/GS) showed similar peaks to CS/GS as apposed to insulin. Stability of samples were evaluated at 1 mg/ml, in MQ water solution, 23 °C, 60% humidity with light exposure for 7 and 28 days. QDs demonstrated instability of functional groups with time. Comparatively, CS/GS, insulin and oral insulin showed minimal changes during stability studies. (**b**) Left panel: 2D [^1^H, ^1^H]-NOESY spectrum of maltose. Right panel: 2D [^1^H, ^1^H]-NOESY spectrum of chitosan and glucose (CS/GS) copolymer. Spectra were collected on a 600 MHz spectrometer with CryoProbe at 298 K. Data were collected and processed using Topspin. NOE depends on the tumbling (rotational diffusion) of the molecule in solution. Small molecules have rapid motions compared to large molecules. Maltose is small in size; therefore, its NOESY cross-peaks are opposite to the sign of the diagonal (diagonal peaks: negative, blue, and off-diagonal peaks: positive, green). NOE cross-peaks are generally small or close to zero for medium-sized molecules (approx. 1–3 kDa). The NOE cross-peaks and diagonal peaks of CS/GS copolymer have the same sign (diagonal and off-diagonal peaks are indicated in blue). Since the molecular weight of CS/GS monomer ~1.8 kDa, we speculate that CS/GS copolymer molecular weight is above 3.6 kDa. The dotted red line shows the NOE connectivity between the NH and CH^3^ moieties of the CS/GS copolymer.

For the protection and controlled release of the insulin payload, we utilized a random polymerization chitosan and glucose copolymer (CS/GS). First, monomers were produced from the hydrolysis of N-deacetylation of chitosan and d-(+)glucose. The solutions were combined in a 2:1 ratio, before condensation polymerization to produce a cationic random copolymer followed by dialysis to collect the 10–30 kDa fraction. CS/GS FTIR and nuclear magnetic resonance (NMR) spectra demonstrated similarities to chitosan and cellulose^31^ with broader –OH functional groups, a reduced CN and a prominent C–O β,4 glycosidic bond at 1,175–1,140 cm^−1^ (Extended Data Figs. 2a and 4a)^32^. Thermogravimetric analysis (TGA) showed CS/GS had a greater mass loss (4.7%) at 50 °C and a new mass loss of 7.6% at 215 °C, indicating that CS/GS is composed of 5:1 CS/GS (Extended Data Fig. 2b).

**Extended Data Fig. 5:**
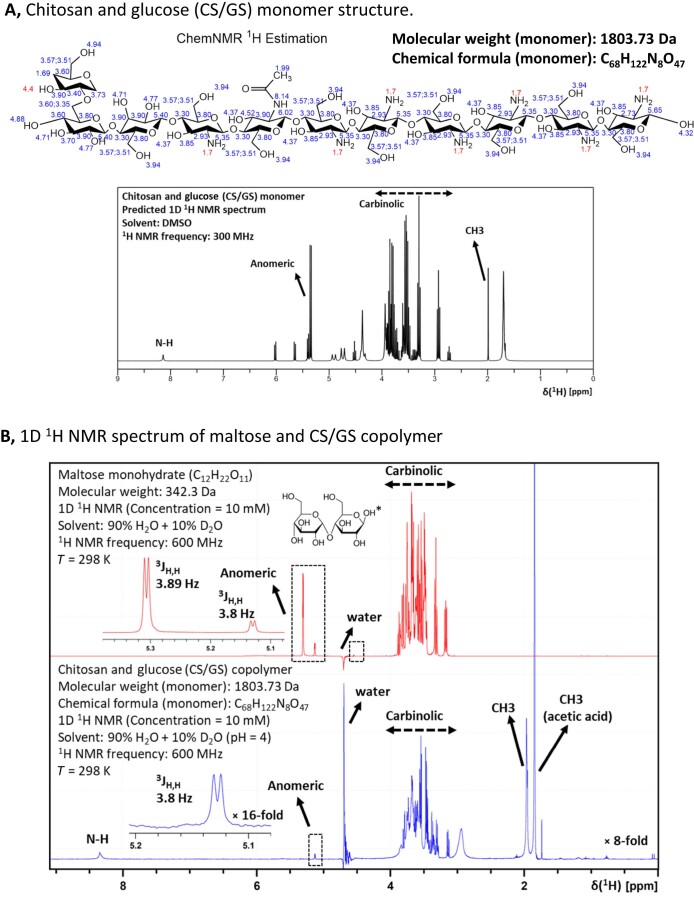
1D ^1^H NMR characterization of chitosan and glucose (CS/GS). (**a**) CS/GS monomer structure. ChemDraw predicted ^1^H chemical shifts are indicated in blue with predicted 1D ^1^H NMR spectrum of chitosan and glucose (CS/GS) monomer. (**b**) 1D ^1^H NMR spectrum of maltose showed two α-anomeric signals were observed ~5.13 ppm and 5.3 ppm (3JH,H ~ 3.8 Hz). The β-anomeric (~4.5 ppm) signal is close to the water and is suppressed due to the water suppression ‘excitation sculpting’ element in the 1D ^1^H NMR experiment. Asterisk indicates the reducing end of the disaccharide. 1D ^1^H NMR spectrum of CS/GS copolymer showed α-anomeric signal at 5.13 ppm (3JH,H ~ 3.8 Hz) is indicated. Spectra were collected using the ‘zgesgp’ Bruker pulse sequence. 1D ^1^H NMR acquisition and processing parameters of maltose and CS/GS copolymer were kept the same. Spectra were collected on a 600 MHz spectrometer with CryoProbe at 298 K. Data were collected and processed using Topspin.

The chemical structure of CS/GS was determined by one-dimensional ^1^H and two-dimensional [^1^H,^1^H] nuclear Overhauser effect spectroscopy (NOESY), NMR and FTIR (Fig. 1b and Extended Data Figs. 2, 4 and 5). The prominence of β-1,4 glycosidic bonds in FTIR was due to the use of deacetylated chitosan (glucosamine monomers) that polymerize via these β-1,4 glycosidic bonds. Glucose monomers typically form α-1,4 or α-1,6 glycosidic bonds when forming glycogen or starch; however, in a long chain, they can also form β-1,4 glycosidic bonds to form cellulose^33^. CS/GS appears to contain both α and β glycosidic bonds and was designed to be susceptible to this form of degradation, with breakdown leading to the release of insulin.

The CS/GS copolymer was coated onto QD-INS by electrostatic attraction between QD-INS and the cationic copolymer at pH 7 due to the negative *ζ*-potential of QD-INS and positive *ζ*-potential of CS/GS at this pH (Extended Data Fig. 1). Hydrogen bonding is probably facilitated at the C terminus of the α and β chains, particularly B21, 25–27 and 30 (Fig. 1b). Mixing these solutions led to the formation of 20.0 ± 1.7 nm CS/GS nanoparticles containing both Ag_2_S QDs and insulin observable under TEM (Fig. 1c). The addition of CS/GS increased the hydrodynamic (HD) diameter measured using dynamic light scattering of QD-INS–CS/GS, with negligible change in the *ζ*-potential (Fig. 1d). Coating with CS/GS abolished the fingerprint markers of insulin in the FTIR and NMR spectra with QD-INS–CS/GS having –OH functional and amine groups (Extended Data Figs. 2a and 4a). This effect was reversible by incubating QD-INS–CS/GS at 37 °C in a pH 3 solution to promote polymer degradation (Extended Data Fig. 2a). The loading capacity of insulin onto QDs at different concentrations showed that the maximum insulin retention was 80%, with 1–2 insulin molecules per QD based on the molar ratios (Fig. 1e).

**Extended Data Fig. 6:**
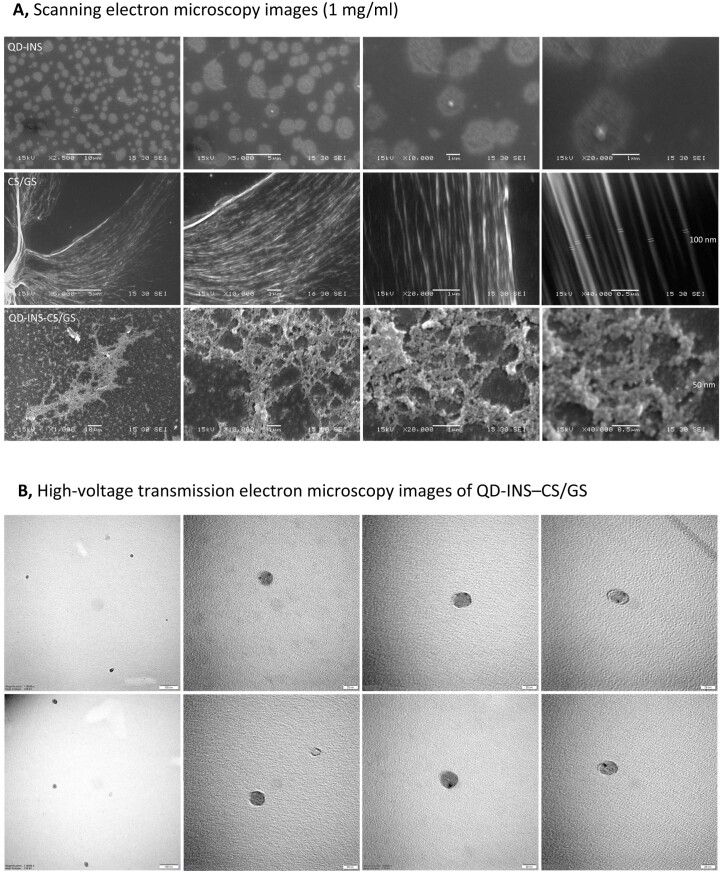
Scanning electron (SEM) and high-voltage transmission electron microscopy (HV-TEM) images. (**a**) SEM images were collected from QD-INS, CS/GS and QD-INS–CS/GS samples. QD-INS demonstrates a disperse drying pattern with minimal samples visible following processing. CS/GS biopolymer was formulated in large chains. Image shows a single chain spread out and dried. Individual chains are visible and approximately 100 nm in diameter. QD-INS–CS/GS demonstrated a different pattern compared to QD-INS. QD-INS–CS/GS showed aggregated samples with spherical structures collectively attached at a single site. (**b**) HV-TEM images showed monodispersed QD-INS–CS/GS compared to SEM samples. Images show both single and double QD expression in a single QD-INS–CS/GS spheroid. Scale bars show in individual images.

CS/GS polymer chains demonstrate more than 1 µm length using scanning electron microscopy (SEM) with a 100 nm fibre diameter (Extended Data Fig. 6a). By comparison, QD-INS–CS/GS was spherical in shape and demonstrated agglomeration. TEM imaging revealed monodispersed particles containing Ag_2_S QDs (Extended Data Fig. 6b).

Increasing the concentration of CS/GS led to a corresponding increase in the HD diameter (Fig. 1f). Interestingly, CS/GS-concentration-dependent effects on HD diameter were also influenced by pH, with low pH increasing the HD diameter and vice versa (Fig. 1f). This effect is probably related to the pH-dependent insolubility and agglomeration of particles (Extended Data Fig. 1). Initial insulin tolerance testing (ITT) in mice demonstrated that the polymer coating was required to reduce blood glucose (BG), highlighting a critical size requirement for efficacy (Fig. 1g).

## Insulin release is mediated by enzymatic cleavage of CS/GS

**Fig. 2:**
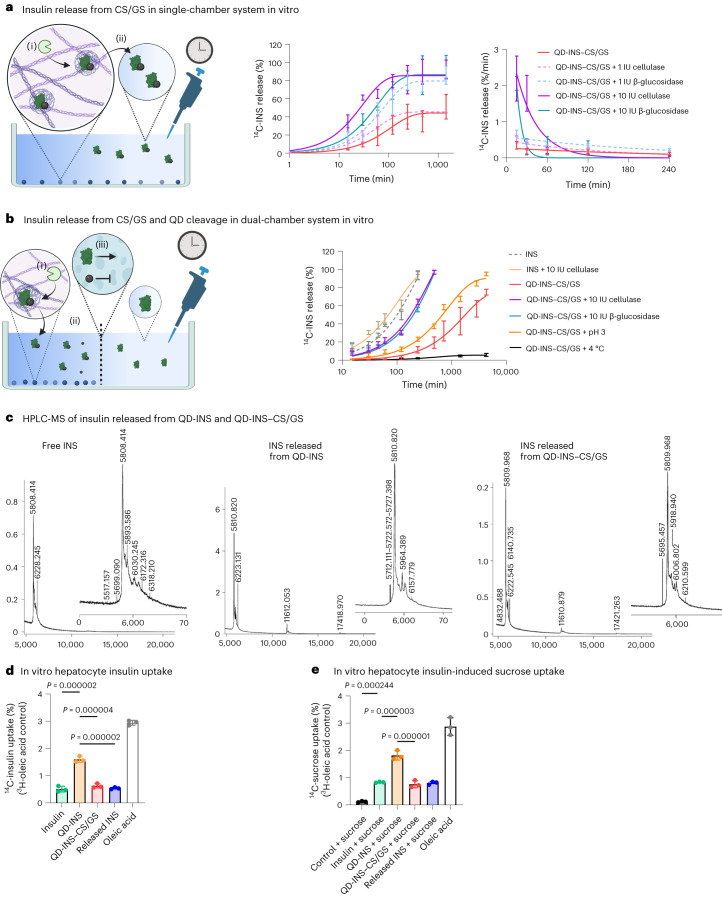
Insulin release from QD-INS–CS/GS. **a**, Increased release of ^14^C-INS in a single-chamber system was promoted by co-incubation with enzymes (for example, β-glucosidase) (i) and to promote the cleavage of CS/GS polymer and release of QD-INS payload (ii). An enzyme dose-dependent effect was observed on the rate of QD-INS release. **b**, Release of ^14^C-INS from QD-^14^C-INS–CS/GS was examined in a dual-chamber system. Cleavage of CS/GS and removal of QDs were promoted by enzyme co-incubation (i). The dual-chamber system was separated by a 10.0 kDa membrane (ii) and allowed only free insulin (5.8 kDa) to pass through the second chamber (iii). Release was strongly dependent on co-incubation with hydrolysing enzymes (cellulase and β-glucosidase), with 50% release within 60 min. Decreased pH and increased temperature also increased the drug release. **c**, High-performance liquid chromatography–mass spectrometry (HPLC–MS) was performed on insulin alone and insulin released from QD-INS and QD-INS–CS/GS. Release was promoted by hydrolysis for 28 days at 23 °C. Analysis shows insulin released from QD-INS and QD-INS–CS/GS has similar spectra to insulin alone, with 87.5% of the insulin released as a monomer. **d**, Uptake of ^14^C-INS alone, in conjugation with QD-INS or QD-INS–CS/GS or after QD-INS–CS/GS release was examined in cultured hepatocytes following a 2-h incubation at 37 °C. Oleic acid was used as a positive control of cellular uptake and activity. Insulin uptake was increased by attachment to QD. **e**, Uptake of ^14^C-sucrose was also investigated following insulin pre-treatment to demonstrate physiological activity. Similar to insulin uptake, sucrose uptake was increased following QD-INS treatment and promoted from QD-INS–CS/GS-released insulin. Data points, mean ± s.d. shown [*n* = 3 independent experiments (**a** and **b**); *n* = 3 biologically independent cells (**d** and **e**) and one-way ANOVA with Tukey’s correction; *α* = 0.05]. **a** and **b** created with BioRender.com.

To examine the release of the insulin payload, we measured carbon-14 (^14^C) insulin (^14^C-INS) release from QD-INS–CS/GS using co-incubation with various hydrolysis enzymes, low pH values and temperatures in hepatocyte cultures. Co-incubation with cellulase or β-glucosidase markedly increased the rate of release, with 50% released within 0.5 h in a single chamber (Fig. 2a). Following this, we utilized a dual-chamber system separated by 10 kDa dialysis tubing to only allow free (non-QD-conjugated) insulin to be detected in the second chamber. Co-incubation with cellulase or β-glucosidase led to a 50% release of insulin within 1 h in the second chamber (Fig. 2b). The rate of release increased with temperature and reduced pH (Fig. 2b), indicating that CS/GS is highly sensitive to enzymatic degradation, particularly β-glucosidase. The released insulin was analysed by mass spectrometry and was primarily a monomer (87.5%) with a minor presence of dimers (11.5%) and trimers (<1.0%) (Fig. 2c). Similar spectral peaks were observed between QD-INS and QD-INS–CS/GS with insulin alone (Fig. 2c).

Insulin and sucrose uptakes were also evaluated in hepatocyte cultures. ^14^C-INS uptake doubled when conjugated with QDs, whereas formulations with QD-INS–CS/GS reversed this effect (Fig. 2d), suggesting that CS/GS interacts with the C terminus of the β chain critical for receptor activation. Pre-treatment of hepatocytes with QD-INS–CS/GS or insulin released from QD-INS–CS/GS highlights that CS/GS must be degraded before insulin action and shows that QD-INS is physiologically active (Fig. 2e).

QD-sized CS/GS particles present several key advantages compared with larger chitosan nanoparticles that have demonstrated the oral mucosal and intestinal delivery of peptides^34,35^. Chitosan in combination with hydroxypropyl methylcellulose phthalate^35^, poly-γ-glutamic acid^36^, alginate^37^, heparin^38^ or polymethacrylic acid/phenethylene glycol^39^ have been shown to produce pH-sensitive nanoparticles (100–300 nm) that promote peptide release at pH 7 and inhibit release at pH 1–3^34,40^. Here we have shown that glucose may also be used, with the added benefits of insolubility across pH 3–7, a QD-sized (<20 nm) carrier for peptides that have rapid intestinal uptake^24^, a surface structure similar to natural cellulose^31^ and sensitivity to hepatic enzyme degradation particularly at a high concentration of β-glucosidase in the liver for the hydrolysis of glycosides and oligosaccharides^41,42^. In addition, these enzymes have a critical role in the breakdown of dietary xenobiotics^43^ and crystalline cellulose^44^ and probably mediate the degradation of the CS/GS polymer in vivo. Glucosidase-responsive nanomaterials have previously been developed to improve the cytotoxic actions of the transported material^45^. These nanomaterials utilized dextran (R-1,4 poly(d-glucose))^46^ or lactose/starch derivatives^47^, similar to CS/GS, which release their payload following degradation.

## Nanocarrier increases human intestinal explant uptake

**Fig. 3:**
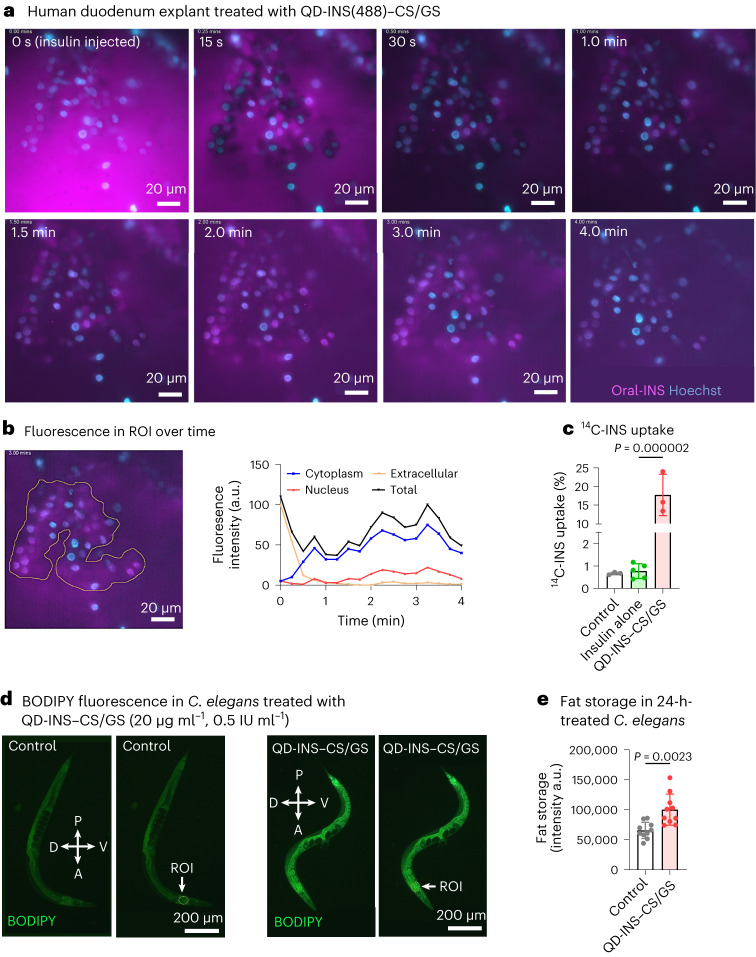
Duodenum explant uptake of QD-INS(488)–CS/GS. **a**, Representative time-lapse imaging of duodenum explants treated with QD-INS-conjugated Alexa Fluor 488 with CS/GS coating (QD-INS(488)–CS/GS). Treatment was received at *t* = 0 s, with images showing unstained tissue/cell outlines at 15 s. At 2–3 min, this tissue/cell area shows localized fluorescence. Scale bar, 20 µm. **b**, Fluorescence was measured in the extracellular space in the tissue/cell area, with subsections of the cytoplasm and nucleus, over the 4-min time-lapse video. Fluorescence intensity was observed to peak at 3.5 min in the cytoplasm followed by a reduction in cell staining. Data points from a single video are provided; the experiment was repeated with five independent tissue samples. Scale bar, 20 µm. **c**, Quantification of ^14^C-INS uptake was evaluated in explants following a 2-h incubation at 37 °C conjugation of insulin with QD-INS–CS/GS leading to a 40-fold increase in insulin uptake compared with insulin alone. **d**, Representative images of BODIPY-stained *C. elegans* were treated with 20 µg ml^–1^ (0.5 IU ml^–1^ insulin) QD-INS–CS/GS for 24 h and *n* = 10 independent animals were analysed. The ROI shows the anterior intestine for fat storage measurements. The compass shows the anterior, posterior, dorsal and ventral regions of worms. Scale bar, 200 µm. **e**, Fat storage was increased in the ROI (anterior intestine) in 24-h-treated QD-INS–CS/GS (0.5 IU ml^–1^) *C. elegans* compared with controls (OP50 fed only). Data points, mean ± s.d. shown (*n* = 3 biologically independent samples (**c**), one-way ANOVA with Tukey’s correction; *n* = 10 biologically independent animals (**e**) and *t*-test with Welch correction; *α* = 0.05).

Intestinal uptake was investigated using human duodenal explants similar to our previous studies in mice^24^. Live imaging of cellular uptake in human duodenum explants (Fig. 3a) demonstrated ex vivo uptake within 2–4 min predominantly in the cytoplasm (70%) (Fig. 3b). Endocytic vesicle formation and exocytosis were observed after 8 min (Supplementary Video 1). Ex vivo human duodenum explants demonstrated a 40-fold greater uptake for ^14^C-INS when formulated with QD-INS–CS/GS (Fig. 3c).

## Insulin nanocarrier promotes fat storage in *C. elegans*

Validation of intestinal uptake was also examined in *Caenorhabditis elegans* (*C. elegans*). The insulin-signalling pathway is highly conserved in *C. elegans*^48^, with this model used for initial screening for oral delivery nanotechnology due to its intestinal barrier and physiological response to insulin^49,50^. Excess insulin inhibits the release and utilization of stored body fat for energy and promotes fat storage^51,52^. We observed that 24-h high dosage of 20 µg ml^–1^ QD-INS–CS/GS mixed in a 1:1 ratio with OP50 *Escherichia coli* (*E. coli*) increased the anterior fat storage compared with controls (Fig. 3d,e).

## Nanocarrier increases bioavailability and efficacy in mice

**Fig. 4:**
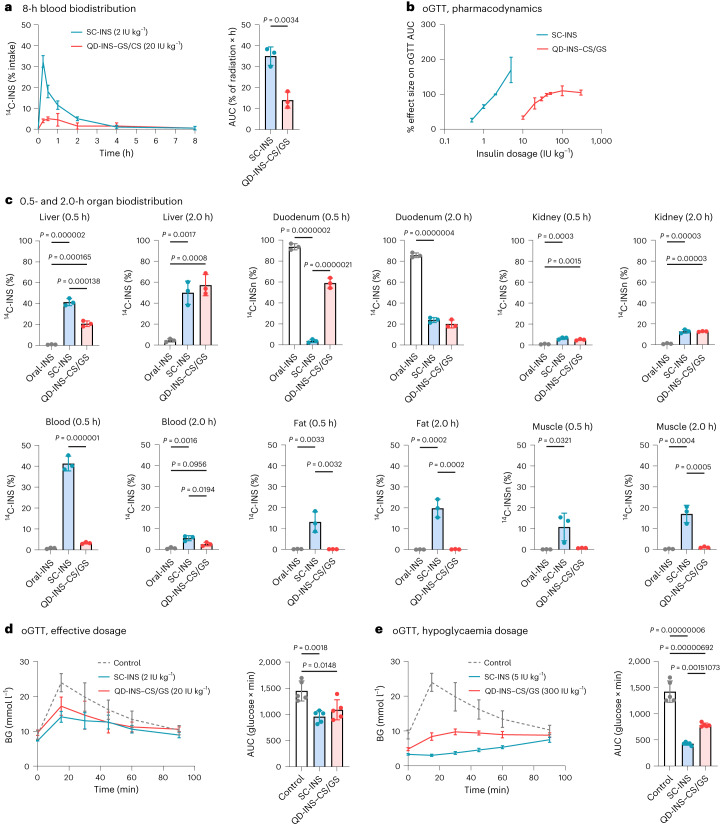
Pharmacokinetics and pharmacodynamics of a single dose of SC-INS, QD-INS–CS/GS and oral insulin alone. **a**, Insulin concentration was measured at 0, 0.25, 0.50, 1.00, 2.00, 4.00 and 8.00 h following a subcutaneous (SC) injection of insulin or oral administration of QD-INS–CS/GS. The AUC demonstrated that the QD-INS–CS/GS biodistribution was 4%. **b**, Pharmacodynamic effect was measured by the effect size in reducing the AUC in oGTTs. Dose–response effects were observed for both SC-INS and QD-INS–CS/GS. Sample oGTTs are shown in **d** and **e**. **c**, Biodistribution of administered ^14^C-INS was measured in the liver, duodenum, kidneys, blood, adipose fat and quadriceps muscle at 0.5 and 2.0 h. Insulin was given by oral administration, SC injection or orally as QD-INS–CS/GS. **d**, oGTTs were performed 15 min post-administration of SC-INS or QD-INS–CS/GS. The AUC demonstrates the equivalent effect between SC-INS (2 IU kg^–1^) and QD-INS–CS/GS (20 IU kg^–1^). **e**, Hypoglycaemia (BG < 2.9 mmol l^–1^) was induced by a high dosage of SC-INS (5 IU kg^–1^). High-dosage QD-INS–CS/GS (300 IU kg^–1^) did not induce hypoglycaemia. Data points, mean ± s.d. shown (*n* = 3 biologically independent animals (**a** and **c**); *n* = 5 biologically independent animals (**b**, **d** and **e**); two-tailed unpaired test (**a**) and one-way ANOVA with Tukey’s correction; *α* = 0.05 (**c**–**e**)).

The biodistribution of subcutaneously injected insulin (SC-INS) and orally administered QD-INS–CS/GS (20 IU kg^–1^) were evaluated using ^14^C-INS. In C57BL/6J mice, the *C*_max_ value of QD-INS–CS/GS was 0.06 IU ml^–1^, *T*_max_ was 0.5 h and *T*_½_ value was 0.5 h. SC-INS (2 IU kg^–1^), by comparison, has a *C*_max_ value of 0.1 IU ml^–1^, *T*_max_ value of 0.25 h and *T*_½_ value of 0.25 h. The bioavailability of oral QD-INS–CS/GS calculated from blood levels was 4% (Fig. 4a).

Distribution in the whole blood, liver, muscles, fat, duodenum, kidneys and spleen was examined for SC-INS, QD-INS–CS/GS and insulin given orally without nanotechnology (Oral-INS). At 0.5 h, SC-INS had a 40%, 40% and 20% distribution in the blood, liver and muscles/fat, respectively (Fig. 4b). By comparison QD-INS–CS/GS had 20% distribution in the liver and 60% in the intestine (Fig. 4c). By 2 h, SC-INS and QD-INS–CS/GS had equivalent 50%, 20% and 10% distribution in the liver, small intestine and kidneys, respectively. SC-INS demonstrated greater distribution in the muscles and fat, whereas QD-INS–CS/GS exclusively targeted the liver. Oral-INS remained in the small intestine.

**Extended Data Fig. 7:**
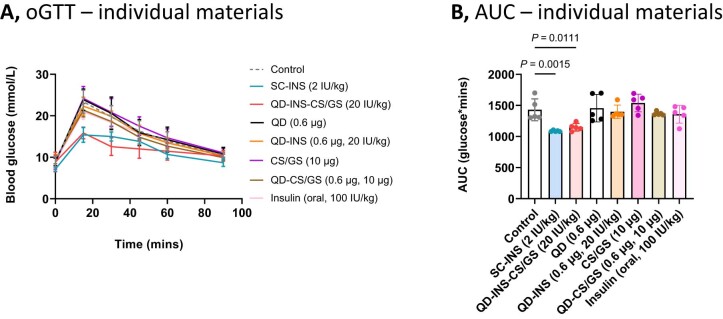
Pharmacodynamics of single dosing with QD-INS–CS/GS constituents. Pharmacodynamic effect was measured by the effect size in reducing the AUC in oral glucose tolerance tests (oGTTs). (**a**) oGTTs were performed 15 min post-administration of SC-INS (2 IU/kg), QD-INS–CS/GS (QD: 0.6 µg, INS: 20 IU/kg, CS/GS: 10 µg), QD alone (0.6 µg), QD-INS (0.6 µg, 20 IU/kg), CS/GS alone (10 µg), QD-CS/GS (0.6 µg, 10 µg) and insulin alone given orally (100 IU/kg). (**b**) AUC demonstrates equivalent effect between SC-INS (2 IU/kg) and QD-INS–CS/GS (20 IU/kg), with no differences compared to controls between any individual constituents or other combinations. Data point, mean ± s.d. shown (n = 5 biologically independent animals, one-way ANOVA with Tukey’s correction; α = 0.05).

Pharmacodynamic effects in C57BL/6J mice were examined using oral glucose tolerance tests (oGTTs) and pre-treatment with SC-INS and oral QD-INS–CS/GS 15 min before oral glucose administration. SC-INS (2 IU kg^–1^) and oral QD-INS–CS/GS (20 IU kg^–1^) demonstrated similar decreases in the area under the curve (AUC) of the oGTT (Fig. 4d). A dose-dependent effect was observed for both injectable and oral insulin (Fig. 4b). High-dose (5 IU kg^–1^) SC-INS induced hypoglycaemia (BG < 3.0 mmol l^–1^), whereas oral insulin did not cause hypoglycaemia even at 300 IU kg^–1^ (Fig. 4e). Individual constituents of QD-INS–CS/GS (QD, 0.6 µg; insulin, 20 IU kg^–1^; CS/GS, 10 µg) were also examined by oGTT. No effect on AUC was observed for individual materials or insulin alone given orally (100 IU kg^–1^) (Extended Data Fig. 7).

**Extended Data Fig. 8:**
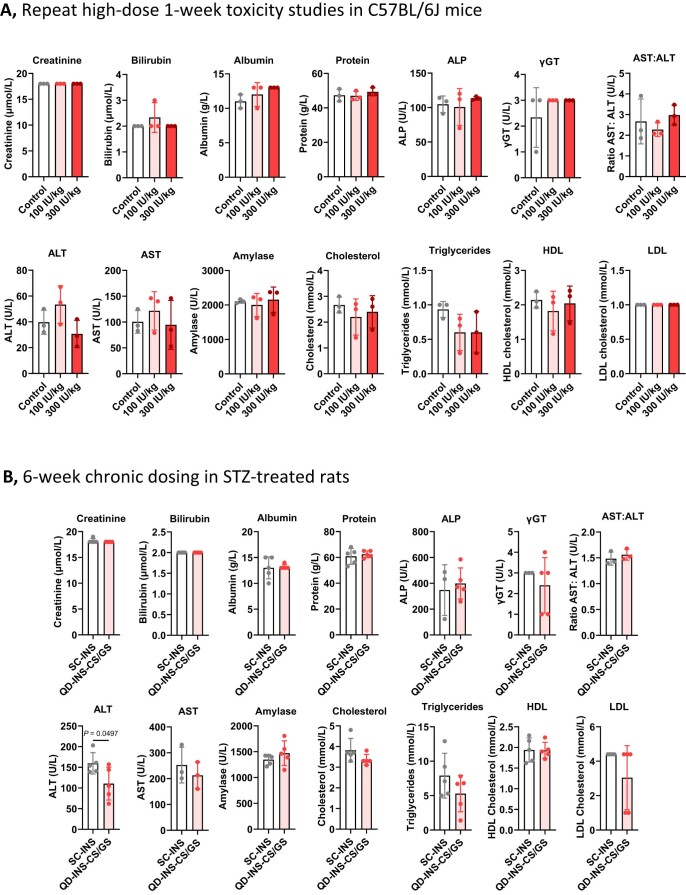
Biochemistry and lipid analysis from repeat dose toxicity studies in C57BL/6 J mice and STZ-treated Wistar rats. (**a**) 12-week-old C57BL/6 J mice (n = 3 per group) were treated for 1 week with repeat-dose QD-INS–CS/GS (treatment days 1, 4 and 7). Dosages were vehicle, 100 IU/kg (0.05 mg/kg QDs) and 300 IU/kg (0.16 mg/kg QDs). Mice were euthanized on day 8 with serum collected and analysed for changes biochemistry and lipids (albumin, amylase, bilirubin, creatinine, protein, yGT, ALP, ALT, AST, cholesterol, triglycerides, HDL and LDL). No changes in biochemistry or lipids were observed between groups. (**b**) Diabetic 20-week-old male Wistar rats (n = 5 per group) (diabetes induced with a single intraperitoneal injection of streptozotocin (STZ) (65 mg/kg) and provided with high-glucose water (10%) for 2 days) were treated daily for 6 weeks with QD-INS–CS/GS. Dosage given was 150 IU/kg/day (0.08 mg/kg QDs); control rats were maintained on 40 IU/kg/day SC-insulin. Rats were euthanized on after 6 weeks with serum collected and analysed as above for changes in albumin, amylase, bilirubin, creatinine, protein, yGT, ALP, ALT, AST, cholesterol, triglycerides, HDL or LDL. No changes were observed in the above blood tests, with QD-INS–CS/GS showing improvement in liver enzyme ALT compared to injectable insulin. Data point, mean ± s.d. shown ((A) n = 3 biologically independent animals, (B) n = 5 biologically independent animals; A: one-way ANOVA with Tukey’s correction, B: two-tailed unpaired *t*-test; α = 0.05)).

**Extended Data Fig. 9:**
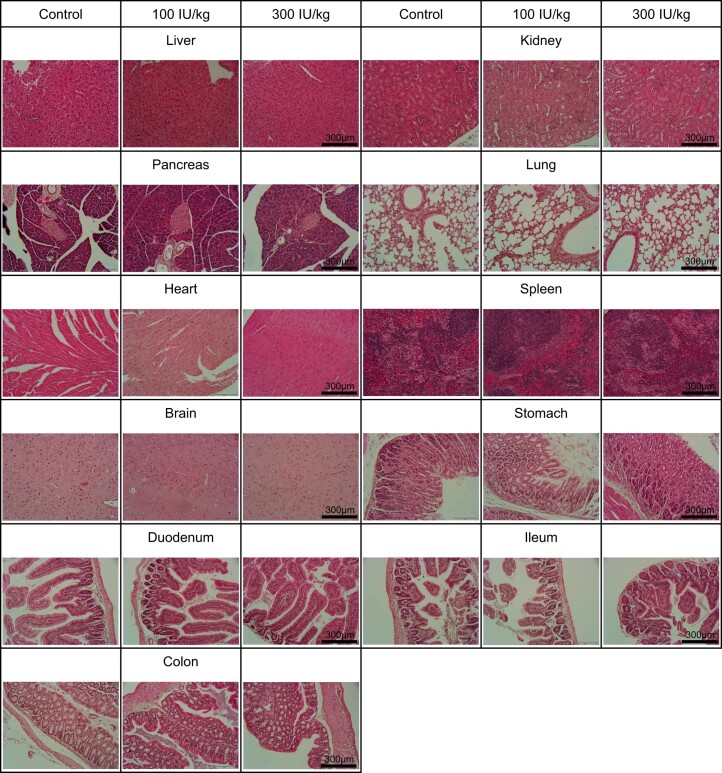
Major organ histology following repeat dose toxicity studies in C57BL/6J mice. Twelve-week-old C57BL/6 J mice (n = 3 per group) were treated for 1 week with repeat-dose QD-INS–CS/GS (treatment days 1, 4 and 7). Dosages were vehicle, 100 IU/kg (0.05 mg/kg QDs) and 300 IU/kg (0.16 mg/kg QDs). Mice were euthanized on day 8 with tissue samples collected for H&E from the liver, kidney, pancreas, lung, heart, spleen, brain, stomach, duodenum, ileum and colon and reviewed for immune cell infiltration and gross histology. No changes in histology were reported.

To investigate the dosage required for QD-INS–CS/GS to induce hypoglycaemia, high-dose treatments were performed in fasted C57BL/6J mice. The minimal dosage required for an adverse hypoglycaemia event was 500 IU kg^–1^ and the lethal dosage (LD_50_) was 1,800 IU kg^–1^. Next, the effect of chronic high-dose treatment was investigated. C57BL/6J mice received either 100 IU kg^–1^, 300 IU kg^–1^ or no treatment via gavage on days 1, 4 and 7, respectively. Compared with controls, no changes in biochemistry or lipids were observed (Extended Data Fig. 8a). Tissue samples collected from major organs showed no changes in inflammatory cell infiltration or necrosis (Extended Data Fig. 9).

Although both silver nanoparticles and chitosan induce toxicity at 0.5–2.0 g kg^–1^ dosages^53,54^, this is 1,000 times greater than dosages that promote adverse/lethal events due to insulin content. Noteworthily, toxicity may be due to the accumulation of QDs over time. However, in a previous study on the toxicity of Ag_2_S QDs (0.36 mg kg^–1^ day^–1^) in 12-week-old C57BL/6J mice treated for 100 days ^23^, these animals showed no retention of Ag^+^ in the liver compared with non-treated control animals, as measured with inductively coupled plasma–mass spectrometry. This study also showed no liver or kidney damage, immune cell activation or circulation^23^.

## Insulin nanocarrier provides efficacy in T1D animal models

**Fig. 5:**
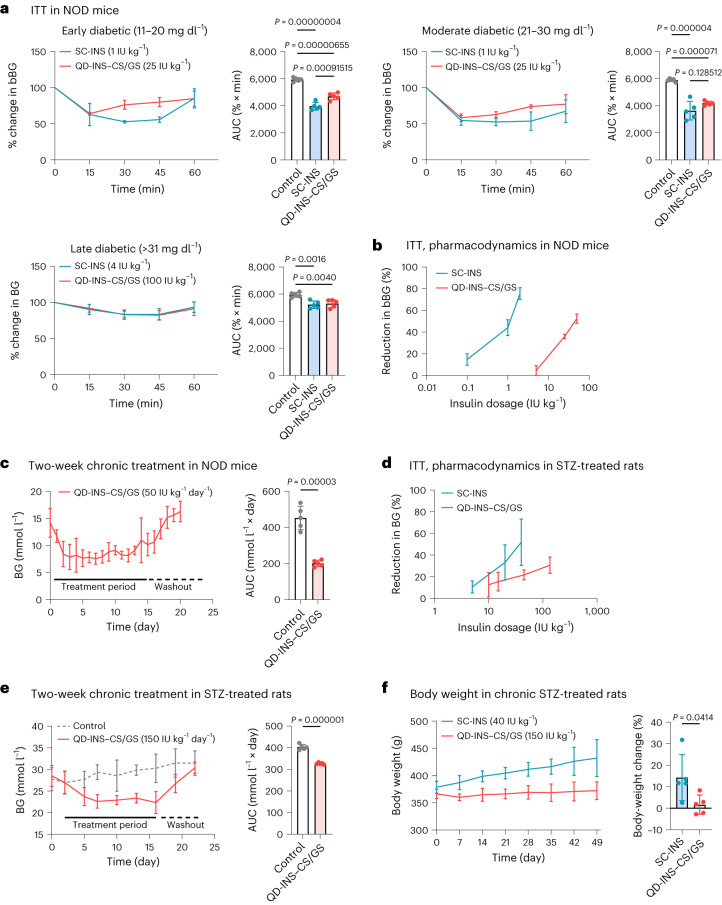
Pharmacodynamics of single-dose and 2-week daily treatment with SC-INS and QD-INS–CS/GS (oral insulin) in NOD mice and STZ-treated rats. **a**, 12–20-week-old NOD diabetic mice were grouped on the severity of their BG levels (early, 11–20 mmol l^–1^; moderate, 21–30 mmol l^–1^; late, >31 mmol l^–1^). Early and moderate diabetic mice were given a single treatment of SC-INS (1 IU kg^–1^) or QD-INS–CS/GS (25 IU kg^–1^), whereas late diabetics were given 4 and 100 IU kg^–1^, respectively. An ITT was performed with BG collected at 0.25, 0.50, 0.75 and 1.00 h. Data show the percentage change in BG and AUC. SC-INS and QD-INS–CS/GS produced similar reductions in AUC and similar time frames to action. **b**, Dose-dependent pharmacodynamic effect was observed in the ITT for NOD mice given either SC-INS or QD-INS–CS/GS. **c**, Two-week treatment with 50 IU kg^–1^ day^–1^ QD-INS–CS/GS was given to NOD mice (BG > 11.1 mmol l^–1^) in their drinking water. BG was recorded daily and was maintained below 11.1 mmol l^–1^ for the duration of treatment. A 7-day washout period showed an increase in BG with the removal of QD-INS–CS/GS. **d**, STZ-treated rats (65 mg kg^–1^, intraperitoneal injection) demonstrated a dose-dependent effect in ITT with SC-INS and QD-INS–CS/GS. **e**, Two-week treatment with QD-INS–CS/GS (150 IU kg^–1^ day^–1^) was given to diabetic STZ-treated rats (BG, >31 mmol l^–1^) in their drinking water; controls were maintained on 40 IU kg^–1^ SC-INS. BG was recorded daily and was maintained between 15 and 25 mmol l^–1^ for the duration of treatment. A 7-day washout period showed an increase in BG with the removal of QD-INS–CS/GS. **f**, Six-week treatment with QD-INS–CS/GS (150 IU kg^–1^ day^–1^) given to diabetic STZ-treated rats demonstrated no change in body weight. Rats maintained on SC-INS (40 IU kg^–1^ day^–1^) showed a 15% increase in body weight over the treatment period. Data points, mean ± s.d. shown (*n* = 5 biologically independent animals; one-way ANOVA with Tukey’s correction (**a**) and two-tailed unpaired *t*-test; *α* = 0.05 (**c**, **e** and **f**)).

The pharmacological effects of SC-INS and QD-INS–CS/GS were compared in single-dose and/or chronic treatment in T1D animal models: non-obese diabetic (NOD) mice (autoimmune-induced T1D) and rats treated with streptozotocin (STZ) (65 mg kg^–1^ single dose) (toxin-induced T1D). In NOD mice, both SC-INS (1 IU kg^–1^) and QD-INS–CS/GS (25 IU kg^–1^) decreased the BG within 15 min and promoted a 40% reduction in ITT AUC (Fig. 5a). Both these examples also showed a dose-dependent reduction in BG with escalating dosage (Fig. 5b). Chronic 2-week dosing with SC-INS or ad libitum administration of QD-INS–CS/GS in drinking water demonstrated that QD-INS–CS/GS maintained BG < 11.1 mmol l^–1^ over the treatment period. This effect reversed following a 7-day washout period (Fig. 5c). SC-INS-treated mice demonstrated reductions in BG 1 h following treatment followed by a return to baseline 8 h post-treatment.

In severely diabetic STZ rats (BG > 30 mmol l^–1^), a similar dose-dependent reduction in BG was observed for injectable and oral insulin (Fig. 5d). Chronic dosing was performed with a 2-week treatment undertaken with SC-INS or ad libitum administration of QD-INS–CS/GS in drinking water. QD-INS–CS/GS maintained a lower BG within 15–25 mmol l^–1^ over the treatment period and this effect was abolished following a 3-day washout period (Fig. 5e). SC-INS-treated rats demonstrated reductions in BG 1 h following treatment followed by a return to baseline within 8 h. Prolonged 6-week treatment demonstrated that SC-INS rats had a 30% increase in body weight, whereas mice treated with QD-INS–CS/GS showed no changes (Fig. 5f). These rats also demonstrated no changes in serum biochemistry or lipids (Extended Data Fig. 8b).

## Insulin nanocarrier provides efficacy in non-human primates

**Fig. 6:**
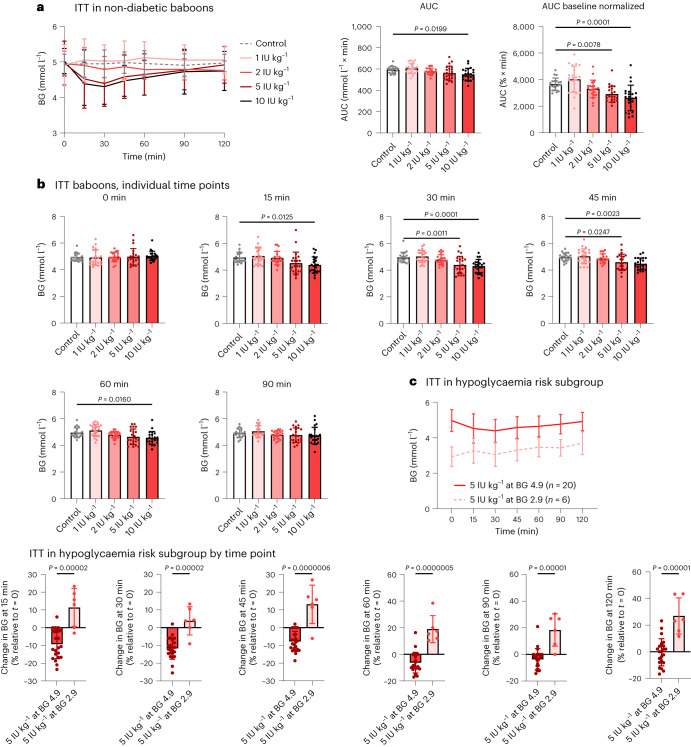
ITT in non-diabetic baboons. **a**, Oral insulin was formulated into 2 g pieces of sugar-free chocolate. Baboons were fasted overnight (16 h) and given a single dosage (0, 1, 2, 5 or 10 IU kg^–1^) of oral insulin. BG measurements were collected at 0, 0.25, 0.50, 0.75, 1.00, 1.50 and 2.00 h time points. ITT AUC was observed to be reduced with 10 IU kg^–1^ QD-INS treatment. Baseline normalization of starting BG also demonstrated reductions in BG. The maximum percentage reduction in blood sugar was observed to be 0%, 5%, 10% and 13% for 1, 2, 5 and 10 IU kg^–1^, respectively. **b**, Individual time points of the ITT. Reduction in BG was observed at 15, 30, 45 and 60 min for baboons treated with 10 IU kg^–1^; 5 IU kg^–1^ showed reductions at 30 and 45 min only. No baboon recorded a BG score below 3.0 mg dl^–1^ for any oral treatment. **c**, Subset of low-BG baboons (2.9 mmol l^–1^) were given 5 IU kg^–1^ oral insulin as above. These baboons showed no change in BG post-treatment. Baboons with a high starting BG (4.9 mmol l^–1^) demonstrated a 10% reduction in their BG. Data points, mean ± s.d. shown (*n* = 20 biologically independent animals (**b**) and one-way ANOVA with Tukey’s correction and unpaired two-tailed *t*-test, *α* = 0.05 (**c**)).

To examine the effectiveness and readiness of QD-INS–CS/GS for clinical applications, we examined non-diabetic baboons from the National Baboon Colony with the BG and toxicity data collection performed independently. Each baboon underwent an ITT after an overnight fast; data were collected following baseline and dosing with 1–10 IU kg^–1^ oral insulin (Fig. 6a). QD-INS–CS/GS treatment with 5 and 10 IU kg^–1^ induced 10% and 13% decreases in BG, respectively, with effects observable at 15–30 min (Fig. 6a). No hypoglycaemia occurred in any baboon given QD-INS–CS/GS even in the subset of baboons with an initial BG of 2.9 mmol l^–1^ given a single 5 IU kg^–1^ dose. The same dose given to baboons with a BG of 4.9 mmol l^–1^ promoted a 10% reduction in BG (Fig. 6c). Blood biochemistry, lipids and haematological assessments following the trial showed no changes outside the reference ranges (Extended Data Table 1). In addition, no adverse events were reported including any episode of hypoglycaemia.

The main finding from this study is the absence of hypoglycaemia observed in mice, rats and baboons. A key aspect of utilizing glucosidase-responsive materials is that these enzymes facilitate saccharide/glycoside/fructose breakdown^41,42,47^, critical roles involved in post-food intake and correlated with glycolytic activity^55^. Recently, it was demonstrated that the β-glucosidase chemical activity directly correlates with glucose concentrations^56^. Together, this suggests that the degradation rate of CS/GS may depend on the glucose/saccharide concentration. Specific hepatic targeting of insulin may also be key to the reduced episodes of hypoglycaemia and reduce the adverse effects of hyperinsulinaemia compared with parenteral administration of animals such as weight gain and insulin resistance^29,57^.

## Conclusion

In conclusion, insulin conjugated to Ag_2_S QDs with a CS/GS polymeric coating was simple to manufacture and reproducible at CSIRO. The product facilitated the oral absorption and hepatic targeting of insulin. In mice, rats and baboons, it demonstrated a dose-dependent effect with a reduced incidence of hypoglycaemia and no toxicity. These studies form the basis for evaluating this formulation in humans, ongoing toxicity and clinical investigations. This technology is designed to be a platform for oral peptide delivery. It is applicable to peptides and proteins that are either liver acting (for example, interferon-α) or other organ specific (for example, the pancreas for glucagon-like peptide-1 agonist (liraglutide)).

## Methods

### Materials

Digital heating mantle (DMS631, Adelab Scientific), silver diethyldithiocarbamate (D93503, Merck), 1-dodecanethiol (471364, Merck), cyclohexane (227048, Merck), ethanol (EtOH; 02851, Merck; 1.00983 Supelco (CSIRO)), acetone (179124, Merck; 1.00014 Supelco (CSIRO)), N_2_ gas (032, BOC), Ar gas (062, BOC), carbogen gas (555, BOC), 3-MPA (M5801, Merck), chitosan (C3646, Merck), glucose (G8270, Merck; 1.00983 Supelco (CSIRO)), glacial acetic acid (A6283, Merck; 1011744 VWR Chemicals (CSIRO)), phosphate-buffered saline (P4417, Merck), EDC (E6383, Merck; E7750, Merck (CSIRO)), NHS (56485, Merck; 24500, Thermo Fisher (CSIRO)), SnakeSkin dialysis tubing 10 kDa (88243, Thermo Fisher Scientific), bovine serum albumin (A7906, Merck), formaldehyde solution 37% (F1635, Merck), sodium bicarbonate (S6014, Merck), sodium carbonate (anhydrous) (PHR1948, Merck), scintillation fluid (Ultima Gold 2x, 6013329, PerkinElmer), 30% H_2_O_2_ (18312, Merck), solvable solution (6NE9100, PerkinElmer), liquid scintillation vials glass (986541, Merck), (^3^H)-oleic acid (O-1518, Merck), ^14^C-INS human (ARC-3146, Bio Scientific), insulin human recombinant (91077C, SAFC), Accu-Chek Performa strips (p-4015630981946, Amcal), Accu-Chek Performa blood glucose meter kit (p-4015630982219, Amcal), STZ (AG-CN2-0046-G001, Sapphire Biosciences), cellulase, enzyme blend (SAE0020, Merck), β-glucosidase from almonds (G4511, Merck), Hoechst 33342 (14533, Merck), Alexa Fluor 488 TFP ester (A37570, Thermo Fisher), TEM grids (71150, Electron Microscopy Sciences), RPMI-1640 (Merck), Percoll (Merck), penicillin–streptomycin (Merck) and foetal calf serum (Merck).

### Ag_2_S QD synthesis

Ag_2_S QD synthesis has been previously described^23,24^. Briefly, 25.6 mg of silver diethyldithiocarbamate and 12 ml of 1-dodecanethiol were mixed with a created N_2_ vacuum followed by an Ar vacuum. The solution was heated to 200 °C (12 °C min^–1^) and held for 1 h. Following synthesis, EtOH was added (88 ml) followed by centrifugation (3,220 g, 0.5 h). Ag_2_S QDs were resuspended in cyclohexane, washed twice with EtOH followed by repeat centrifugation (3,220 *g*, 0.5 h). Aqueous phase transfer was performed with QDs suspended in a 1:1 (v/v) mixture of cyclohexane/acetone. Then, 1 ml of 3-MPA was added per 25 mg of Ag_2_S QDs and mixed at room temperature for 1 h. The QDs were mixed with EtOH and centrifuged at 1,811 g for 5 min. The pellet was redispersed in Milli-Q (MQ) water, washed with EtOH twice and dispersed in MQ water and lyophilized.

### CS/GS copolymer

CS/GS copolymer was produced by combining 5 mg ml^–1^ chitosan with 10% glacial acetic acid in MQ. Under gentle mixing, the solution was heated to 50 °C for 1 h until chitosan dissolved. Then, 2.5 mg ml^–1^ glucose was added to the solution and mixed for 1 h at 50 °C. The solution is allowed to cool to room temperature (0.5 h) and then dialysed in 10 kDa MQ water for 2, 4 and 16 h at room temperature. The solution was diluted to 10 mg ml^–1^ and pH 5 and stored at 4 °C in the dark until use.

### Ag_2_S QD conjugation to insulin

For 1 ml of 1.3 mg ml^–1^ QD-INS (36 IU ml^–1^), combine 17.5 µg Ag_2_S QDs with 1.862 mg EDC and 2.000 mg NHS in 425 µl MQ water and mix for 1 h at 4 °C. Prepare a 100× carbonate–bicarbonate buffer (1.05 mg sodium bicarbonate, 9.27 mg sodium carbonate (anhydrous), 100 µl MQ water). Adjust the solution to pH 9 (add 7.5 µl 100× carbonate–bicarbonate buffer to the QD–EDC–NHS mixture and mix for 2 min). Prepare 2.18 mg insulin human recombinant in 250 µl MQ water with 2 µl of 1 M HCl until dissolved. Add all the dissolved insulin solution slowly to the QD–EDC–NHS mixture (clear solution, if successful) and mix for 4 h. The solution was then dialysed with 10 kDa in MQ for 2, 4 and 16 h at 4 °C. The solution was stored at pH 7 and 4 °C in the dark until use or lyophilized for longer-term storage.

### QD-INS coating with CS/GS

For 1 ml of 1.3 mg ml^–1^ QD-INS–CS/GS, 1 ml clear QD-INS was slowly combined with 20 µl CS/GS polymer stock (10 mg ml^–1^). The clear solution was mixed for 10 min at 4 °C and stored in the dark until use. QD-INS–CS/GS will precipitate in MQ water at a pH of <6, centrifuge at 2,465 *g* for 15 min at 4 °C, remove the supernatant and resuspend at the desired concentration or freeze dry to form a powder (~1.3 mg final volume).

### Radiolabelling

^14^C-INS was conjugated to QDs via EDC/NHS coupling by adding ^14^C-INS with insulin as described above. The samples were prepared at 1,000,000 DPM ml^–1^.

### Immunofluorescent (488 nm) labelling

Conjugation of QDs with an Alexa Fluor 488 TFP ester was performed with 1 mM QDs mixed with EDC and NHS in a ratio of 1:1:1 for 1 h, followed by the addition of carbonate–bicarbonate buffer to raise the pH to 9; 1 mM Alexa Fluor 488 TFP ester was added and incubated at 4 °C for 4 h. The mixture was transferred to dialysis tubing 10 kDa and dialysed for 2, 4 and 16 h at 4 °C in the dark.

### HV-TEM

HV-TEM was performed using the JEOL 1400 instrument at the Australian Centre for Microscopy and Microanalysis. CSIRO samples were measured with a Tecnai 12 TEM (FEI). The operating voltage was 120 kV with images recorded using a FEI Eagle 4k × 4k charge-coupled device camera. Non-biological samples were prepared by evaporative deposition on copper-based TEM grids; CSIRO added a thin carbon film made by using an HHV BT150 benchtop coating system (Ezzi Vision) to their copper-based grids.

### SEM

SEM samples were fixed in 2.5% glutaraldehyde in 0.1 M sodium cacodylate buffer, osmicated, dehydrated in graded EtOH and hexamethyldisilazane, mounted on stubs, sputter coated with platinum and examined using a JEOL 6380 SEM instrument.

### X-ray powder diffraction

Dodecane- and 3-MPA-capped Ag_2_S QDs (10 mg) in Si holders were analysed with a Bruker D8 Advance A25 X-ray diffractometer (Cu Kα radiation (40 kV, 40 mA); Lynx Eye XE-T detector) to obtain the X-ray diffractograms. The samples were scanned over the 2*θ* range of 5°–130° (step size, 0.02°; count, 1.6 s). Phase analyses were performed using the Bruker X-ray powder diffraction search match programme EVA v. 6. The crystalline phases were identified using the ICDD-JCPDS powder diffraction database. Error values in the peak table were calculated based on a diffractometer resolution of 0.005°.

### TGA

Powders (prepared as above, 3–5 mg) were added to a pre-weighed alumina crucible and TGA experiments were conducted under N_2_ at a flow rate of 50 ml min^–1^ on a TA instrument (Mettler Toledo, TGA2). The samples were first blown with N_2_ gas for 0.5 h before heating (25–700 °C, 10 °C min^–1^).

### Zetasizer characterization

HD diameter, polydispersity index and *ζ*-potential were measured using the Zetasizer Nano ZS instrument (Malvern Bioanalytical) at Sydney Analytical, the University of Sydney. CSIRO samples were measured on a Malvern Instruments Zetasizer Nano instrument ZEN3600 with a 4 mW 633 nm He–Ne gas laser. Measurements were performed using 1 µM QD, QD-INS and QD-INS–CS/GS in MQ water that was pH adjusted with HCl and NaOH. HD diameter and polydispersity index measurements were performed using backscatter (173°) data collection with three repeats of 12–15 measurements per sample. All the samples were analysed in disposable folded capillary cells. The *ζ*-potential was measured with five repeats of 10–12 measurements per sample (the maximum setting was 100 measurements). All data were collected with triplicate data points with three independent batches analysed.

### FTIR microscopy

FTIR was performed on a LUMOS FTIR microscope (Bruker) at the vibrational spectrometry facilities of Sydney Analytical, University of Sydney. Ten measurements per sample were performed. Data show the average spectrum across 3,500–700 nm in the attenuated total reflection mode, following atmosphere correction and normalization performed using OPUS 7.0 software (Bruker). An average spectrum was produced from ten individual measurements per material from three independent batches.

### NMR

Individual sample stock was prepared in 90% MQ water and 10% D_2_O at pH 7. For NMR data acquisition, 550 µl samples from each stock were transferred to a 5 mm NMR tube. All one-dimensional ^1^H experiments (water suppression pulse sequence, zgesgp; time domain, 32 K; relaxation delay, 2 s; spectral width, 12.5 ppm; number of scans, 256) were collected on a Bruker 800 MHz spectrometer using a *Z*-gradient TCI probe at 298 K. Two-dimensional [^1^H,^1^H] NOESY spectra were collected on a 600 MHz spectrometer with a cryoprobe at 298 K. Data were collected and processed using TopSpin. The water offset was set to 4.697 ppm. Before Fourier transformation from the time domain to the frequency domain, the time-domain data were zero filled twice, and an exponential decay function with a line-broadening factor of 5 Hz was multiplied. Data were processed using TopSpin. For stability measurements, the samples were stored at a lab temperature of ~295 ± 2 K.

### Insulin loading capacity and release

Loading capacity of insulin was assessed using ^14^C-INS (100,000 DPM) following dialysis to determine the proportion of the remaining or conjugated peptide. Dialysis was performed as described above with a 10 kDa filter; conjugated insulin was unable to pass due to the QD attachment. The concentrations of QDs (0.5–35.2 µg ml^–1^) with fixed insulin (5.0 µg ml^–1^) available were examined. Three individual experiments (batches) were performed per group.

Insulin release was assessed first using a single-chamber in vitro drug release methodology. ^14^C-INS or QD-^14^C-INS–CS/GS (1 mg ml^–1^, 100,000 DPM) was conjugated to the bottom of a 24-well plate coated with 0.2 mg ml^–1^ fibronectin following a 2-h incubation. Wells were washed three times with phosphate-buffered saline and incubated at different temperatures (4 °C or 37 °C) and pH (3 or 7) and with different enzymes (1 or 10 IU cellulase, 1 or 10 IU β-glucosidase). Wells were sampled (10 µl) over 0–72 h. Insulin concentrations were determined using a radiation scintillation counter as described below. Insulin encapsulated in CS/GS was responsive using this approach. Three replicate experiments were performed per group.

A two-chamber in vitro drug release methodology was utilized to separate the ^14^C-INS detection of free versus conjugated (CS/GS bound) insulin. Then, 1 ml QD-^14^C-INS–CS/GS (1 mg ml^–1^, 100,000 DPM) or ^14^C-INS (100,000 DPM) was placed within 10 kDa dialysis tubing and placed in a 1 l vial with MQ water changed at each sampling time (0–72 h). Sampling was collected from both chambers. The experiment was performed at 37 °C with/without different enzymes (1–10 IU cellulase, 1–10 IU β-glucosidase). Insulin concentrations were determined using a radiation scintillation counter as described below. Only free insulin (5.8 kDa) could pass through to the second chamber. Three replicate experiments were performed per group.

### Animal ethics

The mice, rat and baboon programmes were approved by the Animal Welfare Committees of the Sydney Local Health District (SLHD) and were performed in accordance with the Australian Code of Practice for the care and use of animals for scientific research (2013, updated 2021) (Animal Welfare Committee approvals 2018/010, 2019/044 and 2020/021). Care of the animals was conducted in accordance with the Australian National Health and Medical Research Council’s Code of Practice for the Care and Use of Non-Human Primates for Scientific Purposes. All the information provided accords with the Animal Research: Reporting of In Vivo Experiments (ARRIVE) and Declaration of Helsinki guidelines.

### C57BL/6J mice

Three- to four-month-old male C57BL/6J mice were obtained from the Animal Resource Centre in Perth. Animals were housed at the ANZAC Research Institute animal house on a 12-h light/dark cycle and provided with ad libitum access to food and water; 20–25 °C; 50%–60% relative humidity; and bedding, ventilated caging systems and enrichment as per the ARRIVE guidelines.

#### Blood and tissue collection

For biodistribution studies, the mice were not fasted before either a subcutaneous injection of ^14^C-INS (100,000 DPM, 2 IU kg^–1^) or a gavage of QD-^14^C-INS–CS/GS or ^14^C-INS (100,000 DPM, 20 IU kg^–1^). Gavage was performed using an oesophageal gavage needle and delivered in a single rapid dose (100 µl). Blood samples were collected from a tail snip (20 µl) over 0–8 h. Mice were euthanized by a single intraperitoneal injection of 100 mg kg^–1^ ketamine and 10 mg kg^–1^ xylazine in saline at 0.5, 2.0 and 24.0 h post-gavage. Then, 200–250 mg of tissue samples was collected from the liver, spleen, kidneys, lungs, muscles, fat and small intestine (duodenum) along with 100 μl of blood.

#### Hepatocyte cell isolations

The isolation of hepatocyte cells has been previously reported^23^. In brief, C57BL/6J mice were anaesthetized with 10 mg kg^–1^ xylazine and 100 mg kg^–1^ ketamine with liver cannulated via the portal vein and perfused with a Krebs buffer solution (0.142 M NaCl, 6.71 mM KCl, 9.63 mM HEPES and 4.60 mM CaCl_2_) and collagenase at 37 °C. The liver was removed and dissociated in the Krebs buffer solution (without CaCl_2_) at 4 °C. The cells were strained and collected in the Krebs buffer solution (without CaCl_2_ but with 10 g l^–1^ bovine serum albumin). All the centrifugation steps, including the Percoll gradients, were performed at 4 °C. Hepatocytes were isolated by centrifugation at 50 *g*.

#### In vitro hepatocyte endocytosis

Endocytosis assays of ^3^H-oleic acid, ^14^C-INS, QD-INS, QD-INS–CS/GS, insulin released from QD-INS–CS/GS and ^14^C-sucrose were performed using isolated hepatocytes from young mice. Cells were plated at 0.25 × 10^6^ cells per well in a 24-well plate with RPMI media. Cells were washed 2 h after plating and incubated for 8 h before use. Hepatocytes were then incubated with 300 μl of ^3^H-oleic acid and either ^14^C-INS, QD-^14^C-INS or QD-^14^C-INS–CS/GS in DMPI without phenol red for 2 h at 37 °C. To determine the effects of insulin on sucrose uptake in hepatocytes, cells were either untreated or pre-treated with insulin, QD-INS, QD-INS–CS/GS or insulin released from QD-INS–CS/GS (28-day incubation at 23 °C in MQ water) for 2 h at 37 °C. Following pre-treatment, 300 μl of ^3^H-oleic acid and ^14^C-sucrose in DMPI without phenol red was administered for 2 h at 37 °C.

For the analysis of endocytosis of radiolabelled substrates, two fractions were collected: cell media and lysed cells. The cells were lysed with 0.1% sodium dodecyl sulphate solution and collected using a cell scraper. Radioactivity was measured using a scintillation counter (TriCarb 2100TR, PerkinElmer). The proportion of radiation was relative to the total injectate. All the radioactivity studies were performed in triplicate.

#### Biodistribution analysis sample preparation and radiolabelled activity analysis

Tissue and blood samples were weighed, mixed with 1 ml solvable solution and incubated at 60 °C for 4 h to dissolve. Then, 0.2 ml of 30% H_2_O_2_ was added to reduce the dark colour saturation. Radioactivity was measured using a scintillation counter (TriCarb 2100TR, PerkinElmer). Samples were mixed with 10 ml scintillation fluid (five measurements per sample). Baseline measurements were collected from control mice (*n* = 3) that were not treated with radioactive samples. Data were collected and analysed as disintegrations per minute.

#### oGTT

oGTT was performed following a 4-h fast. BG was measured using a handheld glucometer using Accu-Chek proforma strips. Blood was collected following a tail snip. BG was collected prior and for 90 min after an oral bolus of 2 g kg^–1^ glucose solution. The AUC was determined based on the mmol l^–1^ × min.

#### High-dosage toxicity

Three- to four-month-old C57BL/6J mice (*n* = 3 per group) were treated for one week with repeat dose of QD-INS–CS/GS on days 1, 4 and 7. Treatments included vehicle, 100 IU kg^–1^ (0.05 mg kg^–1^ QDs) and 300 IU kg^–1^ (0.16 mg kg^–1^ QDs). Mice were euthanized on day 8, serum collected and analysed for changes in biochemistry and lipid parameters (albumin, amylase, bilirubin, creatinine, protein, yGT, ALP, ALT, AST, cholesterol, triglycerides, HDL and LDL). Tissue samples were collected for haematoxylin and eosin staining from the major organs and reviewed for immune cell infiltration and gross histology.

### NOD/ShiLtJ mice

Ten-week-old female NOD/ShiLtJ mice were obtained from the Animal Resource Centre in Perth. Animals were housed at the ANZAC Research Institute animal house on a 12-h light/dark cycle and provided with ad libitum access to food and water; 20–25 °C; 50–60% relative humidity; and bedding, ventilated caging systems and enrichment as per the ARRIVE guidelines. NOD/ShiLtJ mice were monitored weekly for BG changes, with mice developing T1D between weeks 12 and 24 in 90% of female mice. Diabetic mice were identified following two repeat BG measurements at >11.1 mmol l^–1^.

#### ITT

ITT was performed with/without a 4-h fast. BG was measured using a handheld glucometer using Accu-Chek proforma strips. Blood was collected from a tail snip. BG was collected over 1 h after either a subcutaneous injection of insulin (0–2 IU kg^–1^) or a gavage of QD-INS–CS/GS (0–50 IU kg^–1^). The AUC was determined based on mmol l^–1^ × min.

#### Blood and tissue collection

Then, 200–250 mg of tissue was collected from the liver and pancreas along with 500 μl of plasma blood collected via cardiac puncture. Tissue samples were snap frozen with liquid N_2_ or placed in 4% paraformaldehyde.

### Rats

Ten-week-old male Wistar rats were obtained from the Animal Resource Centre in Perth. Animals were housed at the ANZAC Research Institute animal house on a 12-h light/dark cycle and provided with ad libitum access to food and water; 20–25 °C; 50–60% relative humidity; and bedding, ventilated caging systems and enrichment as per the ARRIVE guidelines.

After a month of acclimation and animal handling, rats were given a single intraperitoneal injection of STZ (65 mg kg^–1^) and provided with high-glucose water (10%) for 2 days. Animals were monitored and BG checked for 10 days post-injection. Diabetic rats were identified following two repeat BG measurements at >11.1 mmol l^–1^.

#### Single-dosage studies

ITT was performed as above; rats were treated with 5–40 IU kg^–1^ SC-INS or 10–150 IU kg^–1^ QD-INS–CS/GS. All the ITT data presented in this manuscript used *n* = 5 mice per group.

#### Chronic dosing studies

Chronic glycaemic management of STZ-treated rats was performed either with daily injections of insulin (40 IU kg^–1^ day^–1^) or with QD-INS–CS/GS (150 IU kg^–1^ day^–1^) in drinking water. Oral insulin was self-administered by rats. Rats were maintained on either treatment for 2–6 weeks, with BG measured morning and evening (prior and 1 h post-injection). Following the treatment period, QD-INS–CS/GS was removed from the drinking water for 3 days to perform a washout.

#### Blood and tissue collection

Following experimentation, the rats were euthanized by a single intraperitoneal injection of 75 mg kg^–1^ ketamine and 10 mg kg^–1^ xylazine in saline. Blood, liver and pancreas tissue samples were collected. Plasma was collected and analysed for biochemistry and lipid parameters as above.

### Non-human primate studies

Eight-year-old male *Papio hamadryas* from the Australian National Baboon Colony (Sydney) were used in this study. Baboons are housed with one male between four and seven females, reflecting the unique social organization of *P. hamadryas*. The outdoor enclosures maintain the troop structure of the colony by allowing visual and vocal contact. They are provided with visual/auditory barriers and shelter, which they can freely access. They have free access to indoor night houses that mimic rock walls and the troop returns to them each night as the central sleeping area.

The husbandry practices at the colony consist of daily cage cleaning, twice daily feeding and annual health screening. Health screening includes physical examination; tuberculin testing; tetanus vaccination; intestinal parasite control; and sample collection for routine biochemical, haematological and microbiological studies. The diet consists of fresh fruit and vegetables, bread, nuts, sunflower seeds and commercial primate pellets. Fresh water is provided ad libitum. Various forms of enrichment are provided to the animals including logs, tree branches, swings, water features and mirrors, all of which are permanent features within or outside the cages. Baboons are also strongly motivated by food, which makes up a large part of additional enrichment given throughout the week. This includes seed and nut tubes, fruit and vegetable ice blocks and food puzzles.

#### ITT

ITT was performed as above with the following modifications. Baboons were fasted overnight (16 h) before ITT experimentation. Baboons received five treatments (once per month) of an escalating dosage of QD-INS–CS/GS (0–10 IU kg^–1^) given via reformulation with sugar-free chocolate squares (2 g). BG measurements were recorded over 2 h.

#### Toxicology

Blood samples were collected by a licensed veterinarian following anaesthesia via intramuscular ketamine (8 mg kg^–1^). Baboon toxicology screening was performed a month prior and post-treatment. Serum samples were prepared and analysed for biochemistry, lipid and haematology parameters.

### Humans

#### Ethics

The programme was approved by the Human Research Ethics Committee of the SLHD and was performed in accordance with the National Statement on Ethical Conduct in Human Research (2007, updated 2018) (Human Research Ethics Committee approval 2022/ETH00387). Patients undergoing a routine diagnostic endoscopy were provided with a letter of introduction, participant information sheet and consent form along with other routine information about their endoscopy. On the day of the endoscopy, the study physician obtained the informed consent. All human samples were deidentified from the researchers, including all information related to the patients. Tissue samples were collected as part of routine endoscopies. Up to two samples were collected from the duodenum from eight patients.

#### Duodenum explant collection

Explant tissue was removed from the stomach atrium and body and the duodenum following routine upper gastrointestinal endoscopy. Tissue samples (2 mm^2^) were placed in a Krebs buffer solution (0.142 M NaCl, 6.71 mM KCl, 9.63 mM HEPES, 4.60 mM CaCl_2_, 2% bovine serum albumin, bubbled with carbogen gas for 0.2 h) at 4 °C until use for radiolabelled uptake or live microscopy experimentation.

#### Explant oral insulin uptake

Explant samples were placed in a 24-well plate with 1 ml of Krebs buffer. Samples were incubated with ^3^H-oleic acid and either ^14^C-INS alone or QD-^14^C-INS–CS/GS for 2 h. The supernatant was removed, and the tissue was washed with warmed phosphate-buffered saline. Tissue samples were dissolved using 1 ml of solvable solution and prepared as stated above for radiolabelled detection.

#### Live microscopy

Explant samples were placed in a 20 ml dish with a purpose-built 10 mm 45°-angled tissue-mounting stand. The tissue samples were fixed with histoacryl glue (B. Braun) for 1 min and bathed in 37 °C Krebs buffer. The tissue samples were stained with Hoechst 33342 (1:1,000 in Krebs buffer) for 20 min at 37 °C and washed before microscopy. Wide-field imaging of explants was performed using a 3i VIVO Spinning Disk IntraVital Confocal microscope. Live imaging was performed using DAPI and FITC channels over a 10-min period. At *t* = 0, QD-INS/488-CS/GS (1:200) was administered. Image analysis was performed using ImageJ software (v. 1.53t, National Institute of Health).

### *C. elegans*

*C. elegans* (EG7941 strain carrying the transgene oxTi396 [eft-3p::tdTomato::H2B::unc-54 3’UTR + Cbr-unc-119(+)]) and *E. coli* strain OP50 were purchased from the Caenorhabditis Genetics Centre. OP50 was used as a food source. The worms were cultivated on a 3 cm plate plated with OP50 at 22 °C. QD-INS–CS/GS (20 µg ml^–1^, 0.5 IU ml^–1^) was mixed with OP50 and fed to *C. elegans* at the L4 stage for 24 h. After 24 h, the worms were stained for lipid droplets, the fat storage organelle in *C. elegans*^52^, using RediStain WormDye Lipid Green (BODIPY stain) as per the manufacturer’s instructions. Worms were anaesthetized using 100 mM levamisole and mounted on a 3% agar pad and imaged using a Leica DMI3000B inverted microscope and a ProgRes CFCool camera. The quantification of lipid staining was performed within the anterior intestine (Fig. 3d, region of interest (ROI) is shown) in OP50 only and OP50 with QD-INS–CS/GS-fed worms (*n* = 10 per group) using ImageJ software.

### Statistics

All the multiple group statistical analyses were performed using one-way analysis of variance (ANOVA) with post hoc Tukey’s correction; the post hoc methods were applied for comparison between multiple groups (GraphPad Prism 8.4.0, GraphPad Software). All the analysis between the groups was performed with a *t*-test with Welch correction. Power calculations were performed as previously described^23^. All data are presented as mean ± standard deviation (s.d.).

### Reporting summary

Further information on research design is available in the Nature Portfolio Reporting Summary linked to this article.

## Online content

Any methods, additional references, Nature Portfolio reporting summaries, source data, extended data, supplementary information, acknowledgements, peer review information; details of author contributions and competing interests; and statements of data and code availability are available at 10.1038/s41565-023-01565-2.

### Supplementary information

Reporting Summary

Supplementary Video 1 Live imaging of duodenum explants treated with QD-INS-conjugated Alexa Flour 488 with CS/GS coating. Explant samples were placed in a 20 ml dish with a purpose-built 10 mm 45°-angled tissue-mounting stand. Tissue samples were fixed with histoacryl glue (B. Braun) for 1 min and bathed in a Krebs buffer at 37 °C. Tissue samples were stained with Hoechst 33342 (1:1,000 in the Krebs buffer) for 20 min at 37 °C and washed before microscopy. Wide-field imaging of the explants was performed using a 3i VIVO Spinning Disk IntraVital Confocal microscope. Live imaging was performed using DAPI and FITC channels over a 10-min period at ×63 magnification. At *t* = 0, QD-INS(488)–CS/GS (1:200) was administered. Video preparation was performed using ImageJ software (v. 1.53t, National Institute of Health).

## Figures and Tables

**Extended Data Table 1:**
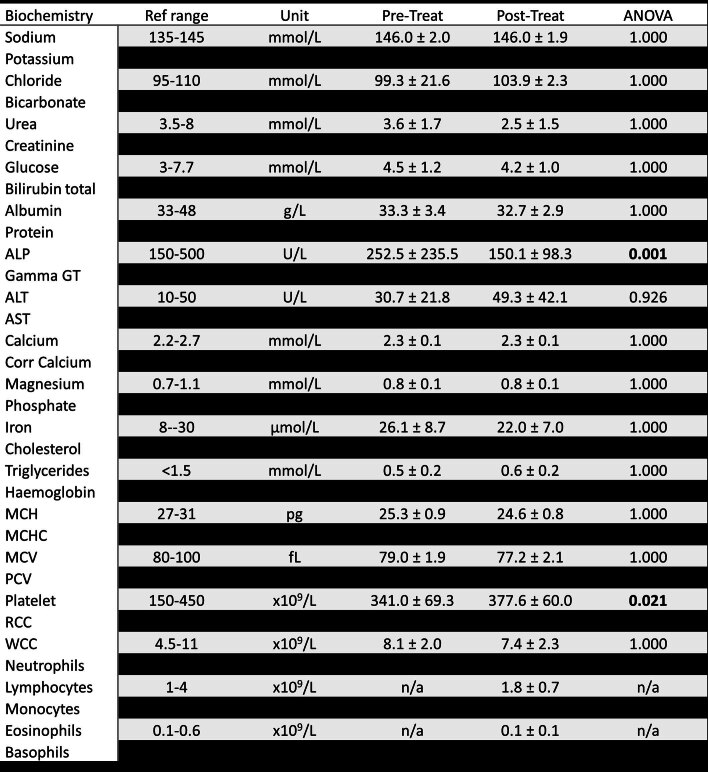
Baboon biochemistry, lipids and haematology following escalating dosage oral insulin treatment

n = 20 biologically independent animals, one-way ANOVA with Tukey’s correction; α = 0.05.

## Data Availability

The datasets generated during and/or analysed during this study, as well as the data that support the plots within this article, the X-ray powder diffraction data and ICDD-JCPDS powder diffraction data, are available from the corresponding authors upon reasonable request.
