# Supplementary material for: Oral nanotherapeutic formulation of insulin with reduced episodes of hypoglycaemia
Source: Nat Nanotechnol. 2024 Jan 2;19(4):534–44. doi: 10.1038/s41565-023-01565-2 (PMC11026164; doi:10.1038/s41565-023-01565-2)
Supplement: Supplementary file 1 — Reporting Summary [file 41565_2023_1565_MOESM1_ESM.pdf]

nature portfolio

Corresponding author(s): Nicholas Hunt, Victoria Cogger

Last updated by author(s): Oct 9, 2023

Reporting Summary

Nature Portfolio wishes to improve the reproducibility of the work that we publish. This form provides structure for consistency and transparency in reporting. For further information on Nature Portfolio policies, see our Editorial Policies and the Editorial Policy Checklist.

Statistics

For all statistical analyses, confirm that the following items are present in the figure legend, table legend, main text, or Methods section.

n/a	Confirmed	
☐	☒	The exact sample size (*n*) for each experimental group/condition, given as a discrete number and unit of measurement
☐	☒	A statement on whether measurements were taken from distinct samples or whether the same sample was measured repeatedly
☐	☒	The statistical test(s) used AND whether they are one- or two-sided<br>*Only common tests should be described solely by name; describe more complex techniques in the Methods section.*
☒	☐	A description of all covariates tested
☐	☒	A description of any assumptions or corrections, such as tests of normality and adjustment for multiple comparisons
☐	☒	A full description of the statistical parameters including central tendency (e.g. means) or other basic estimates (e.g. regression coefficient) AND variation (e.g. standard deviation) or associated estimates of uncertainty (e.g. confidence intervals)
☐	☒	For null hypothesis testing, the test statistic (e.g. *F*, *t*, *r*) with confidence intervals, effect sizes, degrees of freedom and *P* value noted<br>*Give P values as exact values whenever suitable.*
☒	☐	For Bayesian analysis, information on the choice of priors and Markov chain Monte Carlo settings
☒	☐	For hierarchical and complex designs, identification of the appropriate level for tests and full reporting of outcomes
☒	☐	Estimates of effect sizes (e.g. Cohen's *d*, Pearson's *r*), indicating how they were calculated

Our web collection on statistics for biologists contains articles on many of the points above.

Software and code

Policy information about availability of computer code

Data collection

1. Transmission electron microscopy (TEM) images of nanoparticles were obtained with a JEOL 1400 (JEOL, AUS), high voltage transmission electron microscope (HV-TEM) (Australian Centre for Microscopy and Microanalysis, the University of Sydney, AUS). CSIRO samples were measured with a Tecnai 12 TEM (FEI, Eindhoven, The Netherlands) at an operating voltage of 120 kV with images were recorded using a FEI Eagle 4 k × 4 k CCD camera.
2. Scanning electron microscopy was JEOL 6380 Scanning Electron Microscope (JEOL, Tokyo, Japan).
3. X-ray powder diffraction was collected with a Bruker D8 Advance A25 X-ray diffractometer (Bruker, USA), operating under CuKα radiation (40kV, 40mA) equipped with a Lynx Eye XE-T detector was employed to obtain the X-ray diffractograms.
4. Thermogravimetric measurements were performed on a TGA2 Mettler Toledo (TGA2, Switzerland).
5. Hydrodynamic diameter, PDI and ζ potential were measured using a Zetasizer Nano ZS (Malvern Bioanalytical, AUS), at Sydney Analytical, the University of Sydney, AUS. CSIRO samples were measured on a Malvern Instruments Zetasizer Nano instrument ZEN3600 was employed with a 4 mW 633 nm HeNe gas laser.
6. Fourier transformed infrared microscopy was performed on a LUMOS FTIR microscope (Bruker, USA), at the vibrational spectrometry facilities at Sydney Analytical, University of Sydney, AUS.
7. 1H nuclear magnetic resonance was performed on a Bruker 800 MHz spectrometer (Bruker, USA), using a Z-gradient TCI probe at 298 K. 2D [1H,1H]-NOESY Spectra were collected on a 600 MHz spectrometer with CryoProbe at 298 K.
8. Radioactivity was measured using a scintillation counter (Tricarb 2100 TR, PerkinElmer, AUS).
9. Wide-field imaging of explants was performed using a 3i VIVO Spinning Disk IntraVital Confocal microscope (3i, USA).
10. Chemical structures were draw with ChemDraw / ChemOffice v22.0 (revvity signals/ Perkin Elmer, US).

Data analysis	Statistical analysis were performed on Graphpad Prism (version 8.4.0, GraphPad Software, Inc., USA).<br>Image analysis was performed ImageJ software (v1.53t, National Institute of Health, USA).

For manuscripts utilizing custom algorithms or software that are central to the research but not yet described in published literature, software must be made available to editors and reviewers. We strongly encourage code deposition in a community repository (e.g. GitHub). See the Nature Portfolio guidelines for submitting code & software for further information.

Data

Policy information about availability of data

All manuscripts must include a data availability statement. This statement should provide the following information, where applicable:

- Accession codes, unique identifiers, or web links for publicly available datasets
- A description of any restrictions on data availability
- For clinical datasets or third party data, please ensure that the statement adheres to our policy

The datasets generated during and/or analysed during this program are available from the corresponding author upon reasonable request. Including all data that support the plots within this article and other findings of this study including X-ray powder diffraction and ICDD-JCPDS powder diffraction database information.

Human research participants

Policy information about studies involving human research participants and Sex and Gender in Research.

Reporting on sex and gender	No data was collected for sex or gender.
Population characteristics	No patient data collect.
Recruitment	Patients who were considered for or who are booked in for routine diagnostic endoscopy will be approached prior to endoscopy by A/Prof Meng Ngu to determine whether they meet the inclusion/exclusion criteria and to let them know about the study. These criteria were adult patients undergoing routine diagnostic endoscopy who are at low risk for complications from endoscopic biopsies (no antiplatelet or anticoagulant medications, no coagulopathies or platelet disorders) and who can provide informed consent. A letter of introduction, participant information sheet and consent form were provided to the patients along with other routine information about endoscopy at the time of endoscopy booking or at least a week prior to the procedure. A phone number and email contact will be provided if further information is required. On the day of endoscopy, which is a day procedure, the patient will sign the consent form for the study along with the consent form for the endoscopy. The study doctor will be conducting the informed consent for patients that wish to participate. The study staff will not be involved in the general medical management of the patient. Samples were collected from only a single hospital cite with no controls for variability in sex or ethnicity or intestinal health, samples were randomly allocated to groups by researchers that did not engage with patients to reduce selective bias relating to individual patent health.
Ethics oversight	The program was approved by the Human Research Ethics Committee (HREC) of the Sydney Local Health District and was performed in accordance with the National Statement on Ethical Conduct in Human Research (2007, updated 2018) (HREC approval: 2022/ETH00387).

Note that full information on the approval of the study protocol must also be provided in the manuscript.

Field-specific reporting

Please select the one below that is the best fit for your research. If you are not sure, read the appropriate sections before making your selection.

☒ Life sciences ☐ Behavioural & social sciences ☐ Ecological, evolutionary & environmental sciences

For a reference copy of the document with all sections, see nature.com/documents/nr-reporting-summary-flat.pdf

Life sciences study design

All studies must disclose on these points even when the disclosure is negative.

Sample size	Sample size was determined based on our previous work in Hunt et al 2020 and Hunt et al 2021. All experiments were performed at least three times for nanomaterial characterisation or pharmacokinetic studies. We used n=3 as a minimum to obtain statistically meaningful and significant results similar to previous studies (ACS Nano 14 (2), 1492-1507, ACS Nano 15 (3), 4710-4727). Pharmacodynamic effects were measured with at least five biological replicates. n=5 is sufficient to detect significant biological differences with good reproducibility based on our previous experiments (ACS Nano 15 (3), 4710-4727).
Data exclusions	There was no data exclusion.
Replication	In vitro hepatocyte studies were performed in triplicate and were successfully replicated. For all our analyses, we performed experiments across at least 3 biological replicates, all of which were included in our analyses. All attempts at replication were successful.

Randomization	Throughout all studies, samples and animals were randomized into groups.
Blinding	Blinding was used in animal allocation, data collection and analysis. An exception was for experiments involving injected vs gavaged materials the investigator could not be blinded due to the mode of delivery.

Reporting for specific materials, systems and methods

We require information from authors about some types of materials, experimental systems and methods used in many studies. Here, indicate whether each material, system or method listed is relevant to your study. If you are not sure if a list item applies to your research, read the appropriate section before selecting a response.

Materials & experimental systems

n/a	Involved in the study
☒	☐ Antibodies
☒	☐ Eukaryotic cell lines
☒	☐ Palaeontology and archaeology
☐	☒ Animals and other organisms
☒	☐ Clinical data
☒	☐ Dual use research of concern

Methods

n/a	Involved in the study
☒	☐ ChIP-seq
☒	☐ Flow cytometry
☒	☐ MRI-based neuroimaging

Animals and other research organisms

Policy information about studies involving animals; ARRIVE guidelines recommended for reporting animal research, and Sex and Gender in Research

Laboratory animals	3–4-month-old male C57BL/6J mice (Mus musculus), 10-week-old female NOD/ShiLtJ mice (Mus musculus) and 10-week-old male Wistar rats (Rattus norvegicus) were obtained from the Animal Resource Centre (WA, Australia). 8-year-old male baboons (Papio hamadryas) from the Australian National Baboon Colony (Sydney Australia) were used in this study. C. elegans (EG7941 strain carrying the transgene oxTi396 [eft-3p::tdTomato::H2B::unc-54 3'UTR + Cbr-unc-119(+)]) and Escherichia coli strain OP50 were purchased from Caenorhabditis Genetics Centre.
Wild animals	This study did not involve wild animals.
Reporting on sex	Findings for C57BL/6J mice, Wistar rats, baboons and C. elegans apply to one sex. Sex was considered for the use of NOD/ShiLtJ mice as female NOD mice have a higher incidence of type 1 diabetes development compared to male NOD mice. Findings for NOD/ShiLtJ apply to only one sex.
Field-collected samples	This study did not involve samples collected from the field.
Ethics oversight	The program was approved by the Animal Welfare Committees (AWC) of the Sydney Local Health District and was performed in accordance with the Australian Code of Practice for the care and use of animals for scientific research (2013, updated 2021) (AWC approvals: 2018/010 and 2019/044). Care of the animals was conducted in accordance with the Australian National Health and Medical Council's (NHMRC) Code of Practice for the Care and Use of Non-Human Primates for Scientific Purposes. All information provided accords with the ARRIVE and declaration of Helsinki guidelines.

Note that full information on the approval of the study protocol must also be provided in the manuscript.
